# New cell lines expanding the diversity of Ewing sarcoma models

**DOI:** 10.1002/ijc.70172

**Published:** 2025-09-26

**Authors:** Maximilian Kerkhoff, Christiane Schaefer, Wilhelm G. Dirks, Dawid Krzeciesa, Maximilian Bretschneider, Pauline R. Plaumann, Wiebke K. Guder, Arne Streitbürger, Sonja Herter, Heike Peterziel, Ina Oehme, Felina Zahnow, Thomas G. P. Grünewald, Uta Dirksen

**Affiliations:** ^1^ Pediatrics III, West German Cancer Center University Hospital Essen Essen Germany; ^2^ German Cancer Consortium (DKTK) Site Essen Cancer Research Center (NCT) Cologne‐ Essen Germany; ^3^ Faculty of Medicine University of Duisburg‐Essen Essen Germany; ^4^ Leibniz‐Institute DSMZ − German Collection of Microorganisms and Cell Cultures GmbH Department of Human and Animal Cell Lines Braunschweig Germany; ^5^ Department of Medical Oncology, Sarcoma Center, West German Cancer Center University Duisburg‐Essen, Medical School Essen Germany; ^6^ Department of Orthopedic Oncology University Hospital Essen Essen Germany; ^7^ Hopp Children's Cancer Center (KiTZ) Heidelberg Germany; ^8^ Division of Translational Pediatric Sarcoma Research German Cancer Research Center (DKFZ), German Cancer Consortium (DKTK) Heidelberg Germany; ^9^ National Center for Tumor Disease (NCT), NCT Heidelberg, a Partnership Between DKFZ and Heidelberg University Hospital Germany; ^10^ Institute of Pathology Heidelberg University Hospital Heidelberg Germany

**Keywords:** (epi‐)genomics, cell line establishment, drug screen, Ewing sarcoma, transcriptomics

## Abstract

Ewing sarcoma (EwS) is a highly aggressive, malignant, solid tumor of childhood, and adolescence. Most EwS develop in bone, while it originates from connective, adipose, or muscle tissue less frequently. We report the establishment of new human EwS cell lines, all of which carry a t(11;22)(q24;q12) translocation generating the oncogenic transcription factor EWSR1::FLI1. Sequencing of the chimeric mRNAs indicated genomic DNA breakpoints localized in intron 8 of the *EWSR1* gene and three different introns of the translocation partner gene *FLI1*. While three EwS cell lines carry the *EWSR1::FLI1* fusions of ex7/ex6 (type I) or ex7/ex5 (type II), the *EWSR1::FLI1* fusion variant ex7/ex7 (type IV) is described for the first time in a continuous EwS model. The cell lines presented genomic, epigenomic, and transcriptomic stability over a period of 6 months, though some variations in the chromosomal aberrations were observed in one cell line. *TP53*, *STAG2*, and *CDKN2A/B* mutations were the most frequent and most relevant mutations in our cell line panel. The *TP53* mutational status seemed to have the biggest impact on drug sensitivity profiles. The new EwS models presented here may help to identify small molecule inhibitors that act directly on *EWSR1::FLI1* fusion proteins or uncover other genetic vulnerabilities of the altered epigenome and transcriptome in EwS, which would contribute to a better understanding of Ewing sarcoma tumorigenesis.

## Graphical abstract

What's new?

A chromosomal translocation between the *EWSR1* and *ETS* genes is the key oncogenic driver in Ewing sarcoma. *EWSR1::FLI1*, the most frequent fusion, is subdivided into different fusion types with unclear prognostic relevance. Here, the authors describe the establishment and characterization of four new, intrinsically immortal, patient‐derived Ewing sarcoma cell lines, including the first described cell line with an *EWSR1::FLI1* type IV translocation. The new models may help identify small molecule inhibitors that act directly on *EWSR1::FLI1* fusion proteins or other genetic vulnerabilities within the altered epigenome and transcriptome, potentially contributing to a better understanding of Ewing sarcoma tumorigenesis.

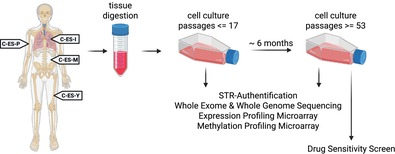

**LIST OF ABBREVIATIONS**
CNV: copy number variant
DBD: DNA‐binding domain
DEG: differentially expressed gene
DMEM: Dulbecco's modified Eagle medium
DMSO: dimethyl sulfoxide
dNTPs: deoxyribonucleoside triphosphate
DSP: drug sensitivity profile
DSS: drug sensitivity score
EDTA: ethylenediaminetetraacetic acid
EwS: Ewing sarcoma
FBS: fetal bovine serum
f‐TRAP: fluorescent telomeric repeat amplification protocol
gDNA: genomic DNA
GSEA: gene set enrichment analysis
hMSC: human mesenchymal stem cells
IMDM: Iscove's modified Dulbecco's medium
MNV: multi‐nucleotide variant
MSI: microsatellite instability
MSS: microsatellite‐stabile
ORA: overrepresentation analysis
PBS: phosphate buffered saline
PC: principal component
PCA: principal component analysis
PCR: polymerase chain reaction
PDT: population doubling time
PRD: proline‐rich domain
RPMI: Roswell Park Memorial Institute Medium
SNV: single nucleotide variant
STR: short tandem repeat
TA: telomerase activation
WES: whole exome sequencing
WGS: whole genome sequencing

## 1 INTRODUCTION

Ewing sarcoma (EwS) is a rare bone and soft tissue malignancy that mainly occurs in children, adolescents, and young adults. Despite intensive multimodal treatment, including high‐dose chemotherapy with stem cell transplantation and the use of targeted agents, one third of all patients with primary non‐metastatic disease develop a relapse with an unfavorable survival rate of <30%.^1^, ^2^, ^3^

EwS is characterized by a chromosomal translocation leading to the formation of an oncogenic transcription factor involving *EWSR1* and a member of the *E‐twenty‐six* (*ETS*) family transcription factors, predominantly represented by *FLI1*. The breakpoint regions may differ, leading to other translocation subtypes with unknown prognostic relevance. Secondary genetic aberrations that facilitate or enable the maintenance of transformed EwS cells are rare and include mutations in *TP53* and *STAG2*, deletions of chr9p and *CDKN2A*, and amplifications (or gains) of chromosomal segments in chr8, chr2, chr1q, or chr20. Extensive analyses have shown that mutations in *TP53* and/or *STAG2* have a poor prognostic value and are noted to have a higher incidence of treatment resistance.^4^, ^5^

Investigating the cell type of origin transformed by *EWSR1::FLI1* proved to be challenging due to the unavailability of suitable animal models.^6^ Recently, advances have been made in zebrafish, supporting a neural crest origin.^7^ Nevertheless, other comprehensively described models are urgently needed. Patient‐derived cancer cell lines are useful tools in the investigation of the clinically heterogeneous group of EwS, as they capture a wide range of patient characteristics such as disease state and progression, translocation subtypes, treatment response, and mutational burden.^8^, ^9^, ^10^

In this article, we describe the establishment and characterization of four new, intrinsically immortal, patient‐derived EwS cell lines. Genomic, transcriptomic, and epigenomic profiling were performed. In combination with a customized drug library screen, we provide extensively screened and described EwS cell lines. Furthermore, we present the first described EwS cell line with an *EWSR1::FLI1* type IV (exon 7/exon 7) translocation.

## 2 MATERIALS AND METHODS

### 2.1 Cell culture and establishment of new EwS cell lines

Solid tumor samples were minced and incubated in 5 mL of 0.05% Trypsin–EDTA (Fisher Scientific; Cat. No. 25300054) for 20–60 min at 37°C and 300–400 rpm on an orbital shaker until the solution turned turbid. After tissue dissociation, the solution was filtered through a MACS® SmartStrainer with a mesh size of 100 μm (Miltenyi Biotec; Cat. No. 130‐098‐463) to remove residual tumor clumps. Following centrifugation of the flowthrough at 250 rcf for 5 min, the supernatant was discarded. Cell pellets of red color were resuspended in 5 mL Gibco™ ACK lysing buffer (Fisher Scientific; Cat. No. A1049201), incubated for 5 min at RT, and centrifuged to remove erythrocytes. The cell pellet was resuspended in 7 mL of Dulbecco's modified Eagle medium (DMEM, high glucose, GlutaMAX™ Supplement; Fisher Scientific 10566016) supplemented with 10% (final concentration) fetal bovine serum (FBS; Merck/Sigma‐Aldrich; Cat. No. F7524) and transferred into a T25 flask (Sarstedt; Cat. No. 83.3910.002) that was previously coated with rat collagen. The cells were monitored daily for the first 1–2 weeks, and conditions may have been adapted (e.g., media exchanges, expansion to bigger flasks or additional FBS).

Liquid tumor samples (in case of ascites) were transferred into 50 mL centrifugation tubes and centrifuged at 250 rcf for 5 min. The resulting cell pellets were resuspended in DMEM + 10% FBS, pooled, and transferred into a T25 flask.

For the first 2–5 passages, cells were split 1:2 by trypsinization and later adapted to higher split ratios, according to their proliferation behavior. There were no immortalizing factors added to the cells. Due to the low proliferation rate of C‐ES‐I in DMEM + 10% FBS, C‐ES‐I was cultured in Iscove's modified Dulbecco's medium (IMDM) + 10% FBS for optimal growth conditions in all subsequent experiments. The patient‐derived cells did not show any signs of contact inhibition and were able to proliferate further, even when confluency was reached.

For the coating, 1.6 mL rat collagen (CellConcepts; Cat. No. Z‐17C03‐C) and 46.4 mL of 0.1 M acetic acid were mixed, and 700 μL of this solution was added into a T25 flask (the volume was adapted for other flasks or dishes according to surface area). The flask was rocked and lightly tapped to ensure even distribution of the coating solution on the flask bottom. The flask was left open overnight in a flow cabinet to allow the coating to dry.

The human mesenchymal stem cell (hMSC) line 70.2 was kindly provided by PD Dr. Bernd Giebel (Institute for Transfusion Medicine, Essen, Germany). STA‐ET‐1 and EW‐7 were cultivated in RPMI 1640 (Gibco) with 10% fetal bovine serum (FBS) at 37°C and 5% CO_2_; hMSC 70.2 was cultivated in DMEM supplemented with 10% FBS and basic fibroblast growth factor at a final concentration of 3 ng/mL (R&D Systems; Cat. No. 233‐FB). All cell lines were subjected to regular testing for mycoplasma contamination.

### 2.2 Testing for mycoplasma contamination

Before splitting cells, 100 μL of medium was harvested from each cell culture flask and was boiled at 95°C for 10 min. The denatured samples were cooled down on ice. A master mix was prepared, containing 5 μL 2xMyTaq HS Red Mix (Cat. No. BIO‐25047), 2.5 μL dH_2_O, 0.5 μL 10 μM forward‐primer (5′‐GGGAGCAAACAGGATTAGATACCCT‐3′), 0.5 μL 5 μM reverse‐primer 1 (5′‐TGCACCATCTGTCACTCTGTTAACCTC‐3′), and 0.5 μL 5 μM reverse‐primer 2 (5′‐TGCACCATCTGTCACTCCGTTAACCTC‐3′) per reaction. One microliter of sample was added to 9 μL of master mix. An additional reaction was set up with a positive control sample. After an initial denaturation at 94°C for 2 min, 30 cycles of 94°C for 20 s, 60°C for 20 s, and 72°C for 30 s were run, followed by 2 min of final elongation at 72°C. PCR products were visualized via agarose gel‐electrophoresis.

### 2.3 Fluorescent telomeric repeat amplification protocol assay

The fluorescent telomeric repeat amplification protocol (f‐TRAP) assay is a recently published method for the determination of telomerase activity in mammalian cells and tissue samples on a fluorescence basis.^11^ The assay involves extension of a fluorescently labeled bait primer, PCR amplification of the extensions, and detection of telomerase products by sensitive capillary electrophoresis, which rapidly and robustly analyzes the presence or absence of telomerase activity.

### 2.4 PCR and Sanger sequencing

Cell line RNA was isolated utilizing the QiaShredder and RNeasy Mini Kit (Qiagen; Cat. Nos. 79654 and 74104) following the manufacturer's instructions. For reverse transcription, 1 μg of RNA was incubated with random hexamers, M‐MLV reverse transcriptase, and RNAseOUT™ according to the manufacturer's protocol (Promega; Cat. No. M1701).

Amplification of *EWSR1::FLI1* transcripts was carried out by conventional PCR with respective primers binding in exon 7 of *EWSR1* and in exon 9 of *FLI1* (for primer sequences, see Table 1). PCR reactions of 50 μL contained 10 μL 5× buffer, 1 μL of 10 μM dNTPs, 2.5 μL of 10 μM primer each, 1.5 μL DMSO, 0.5 μL Phusion HotStart II polymerase (Fisher Scientific; Cat. No. F530S), and 1 μL sample cDNA. The specific cDNA was amplified using the following PCR conditions: 98°C (1 min), 30× [98°C (10 s), 50–60°C (30 s), 72°C (30 s)], and final elongation at 72°C (10 min).

**TABLE 1:** Primer Sequences for amplification and Sanger sequencing.

Name	Sequence (5′–3′)
EWSR1::FLI1_for	TCCTACAGCCAAGCTCCAAGTC
EWSR1::FLI1_rev	GTTGGCGCTGTCGGAGAGC
EWSR1::FLI1_seq_for	CAGAGCAGCAGCTACGGGCA
EWSR1::FLI1_seq_rev	TTGGCGCTGTCGGAGAGC
TP53_for	TGGCAGCCAGACTGCCTTCC
TP53_rev	CAGTGGGGAACAAGAAGTGG
TP53_seq_for	GGCCATCTACAAGCAGTCAC
TP53_seq_rev	ATTTCCTTCCACTCGGATAAGATG

*Note*: Primers with “seq” in their name were utilized for Sanger sequencing; others for amplification.

PCRs for *TP53* (for primer sequences, see Table 1) were performed with the following conditions: 98°C (30 s), 35× [98°C (20 s), 56°C (20 s), 72°C (30 s)] and 72°C (3 min).

For Sanger sequencing, the corresponding PCR products were purified with the GeneJet PCR purification kit (Fisher Scientific; Cat. No. K0701) and 20–40 ng/μL were mixed with 4 μM (final concentrations) of the respective sequencing primer (for primer sequences, see Table 1). Sanger sequencing was conducted at Microsynth Seqlab GmbH (Göttingen, Germany).

### 2.5 Western blot

For sample preparation, cell populations were trypsinized, washed in PBS, and lysed as described previously.^12^, ^13^ All washing and antibody incubation steps were performed in Net‐G buffer (1.5 M NaCl, 50 mmol/L EDTA, 500 mmol/L Tris, 0.5% Tween 20, and 0.4% gelatine). The primary antibody to detect FLI1 (#ab15289) was acquired from Abcam (Cambridge, Great Britain); β‐Actin (#sc‐47778) antibody was from Santa Cruz Biotechnology (Santa Cruz, CA, USA). Secondary antibodies were horseradish‐peroxidase conjugated anti‐mouse IgG (#C7076) from Cell Signaling Technology and anti‐rabbit IgG (#554021) from BD Pharmingen.

### 2.6 Proliferation rate determination

Viable cells were determined by Trypan blue exclusion using Gibco™ Trypan Blue solution 0.4% (Fisher Scientific; Cat. No. 15250061) and were counted in a Neubauer counting chamber under the microscope (20× magnification) after 48, 72, or 96 h. The doubling time was calculated by the following formula:

$$\text{doubling time}=\frac{t_{1}-t_{0}}{\log_{2}\frac{n_{1}}{n_{0}}}$$

with the cell number *n*_0_ at starting timepoint *t*_0_ and the cell number *n*_1_ at end timepoint *t*_1_.

### 2.7 Sample preparation for STR profiling

Authentication of cell lines was conducted by short tandem repeat (STR) profiling at Multiplexion GmbH (Heidelberg, Germany), in accordance with the globally established standard guidelines of ANSI/ATCC ASN‐0002‐2011. For PCR reaction, gDNA was isolated from corresponding cell lines or donor material by either utilizing the QiAMP DNA Mini Kit (Qiagen; Cat. No. 51304) or NucleoSpin™ Tissue Kit (Macherey Nagel; Cat. No. 740952.50) according to the instructions provided by the manufacturers. Genomic DNA was analyzed with the Applied Biosystems™ AmpFLSTR™ Identifier™ Plus PCR Amplification Kit with a panel of 16 selected markers. Electrophoresis was performed on an ABI capillary sequencer. Uniqueness of each cell line was confirmed by comparison using the DMSZ and ATCC databases.

### 2.8 BAT‐26 and BAT‐25 analysis

Primers used to amplify BAT‐26 and BAT‐25 were as described previously.^14^ Antisense BAT‐26 and sense BAT‐25 primers were labeled with the fluorescent probes FAM and NED, respectively. Individual or multiplex PCR was performed using the following conditions: denaturing at 94°C for 5 min, 35 cycles of denaturing at 94°C for 30 s, annealing at 55°C for 30 s, and extension at 72°C for 30 s. This was followed by an extension step at 72°C for 7 min. PCR products were separated using an ABI Prism 3100 16‐capillary genetic analyzer (Applera France, Courtaboeuf, France). Due to their repetitive nature, BAT‐26 and BAT‐25 PCR products are obtained as a succession of peaks differing by 1 bp units. Genescan and Genotyper 2.1 software (Applera France) were used to integrate the areas of each individual peak in arbitrary units. We calculated the percentage of experimentally obtained shortened BAT alleles by adding values obtained for each peak in the corresponding area, as compared to the total values of all peaks (shortened plus normal). The same calculation was also done for normal alleles for each amplified DNA sample.

### 2.9 Sample preparation for WES, WGS, and methylation array

DNA sample preparation was carried out after cell seeding and continuous culturing for 96 h as previously described.^15^ For subsequent analyses, 20 ng/μL gDNA for WES and WGS respectively 18–100 ng/μL for methylation analyses, were used. The quality requirements for the NGS data have been met (Table S1).

### 2.10 CNV calling

Whole‐genome and whole‐exome sequencing was performed at the Next Generation Sequencing Core Facility of the German Cancer Research Center (DKFZ, Heidelberg, Germany). WGS data were mapped to the GRCh38 genome assembly (homo_sapiens.grch38.111.gtf) using the Varlociraptor pipeline^16^ with the—until option up to the node apply_bqsr. Non‐canonical chromosomes were filtered out prior to copy number variations (CNV) analysis. The pipeline cnvkit^17^ was used to determine CNVs and ploidies of the cell lines. The heatmap was generated with the –d option for de‐emphasizing low‐amplitude segments.

### 2.11 Variant calling

WES data was mapped to GRCh38 genome assembly and analyzed via the variant calling pipeline Varlociraptor, able to call SNVs, MNVs, indels, arbitrary replacements, inversions, duplications, haplotype blocks, and break ends.^16^ We utilized a single sample germline scenario (due to the lack of normal controls) and treated every sample independently for variant calling (https://varlociraptor.github.io/varlociraptor-scenarios/scenarios/single-sample-germline-prior/). A subset of top hits was generated by filtering for hits that were contained in a specific gene list (https://www.oncokb.org/cancer-genes) and had an impact of “high” or a clinical significance of “pathogenic,” “likely pathogenic,” or “conflicting interpretations of pathogenicity”. These top hits were plotted with the “Waterfall()” function from the GenVisR package (version 1.36.0) for R.^18^

### 2.12 Sample preparation for Affimetrix microarray

For gene expression analysis, EwS cell lines were seeded and lysed. The isolated RNA was reverse transcribed as previously described.^15^ The isolated RNA was subjected to microarray analysis using the human Affymetrix Clariom D platform, which was performed at the Next Generation Sequencing Core Facility of the German Cancer Research Center (DKFZ, Heidelberg, Germany).

### 2.13 Gene expression analysis

Gene expression profiles were examined using microarray (Affymetrix Clariom D Human Array). We analyzed the microarray data in R studio as described by others.^19^ Gene Set Enrichment Analysis (GSEA) was performed using the msigdbr package (version 7.5.1). For overrepresentation analysis (ORA), the topGO package (version 2.56.0) was utilized.

### 2.14 DNA methylation analysis

An illumina infinium methylation EPIC v2.0 beadchip was utilized. Data analysis was performed as described by others.^20^

### 2.15 Drug sensitivity profiling

Drug screens with metabolic activity read‐out were performed as previously described.^21^ In summary, cells were tested against a library of 80 drugs (including both targeted therapies and chemotherapeutics). Ready‐to‐use 384‐well U‐bottom plates were prepared with every drug in a concentration range spanning five orders of magnitude, with each condition tested in duplicate. Control wells were included to represent maximum effect (100 μM benzethonium chloride) and minimum effect (DMSO). The plates were prepared at the FIMM High Throughput Biomedicine Unit (FIMM, HiLIFE, University of Helsinki, Finland) and stored in an oxygen‐ and moisture‐free environment at room temperature using a San Francisco StoragePod system (Roylan Developments, Fetcham Leatherhead, UK) until further use. For each tested cell line, 1000 cells per well were seeded into the plates, followed by 72 h of incubation. To assess ATP levels, 15 μL of Cell Titer‐Glo 2.0 reagent (Promega, Madison, WI) was added to each well, and luminescence was measured using a PHERAstar FSX plate reader (BMG Labtech, Ortenberg, Germany). The raw luminescence data were analyzed with the web‐based Shiny application iTreX to calculate drug sensitivity scores (DSSasym^22^).

## 3 RESULTS

### 3.1 Patient information

The tumor samples were obtained from pediatric and young adult EwS patients at the time of diagnosis or relapse. All patients presented with an *EWSR1::FLI1* translocation, which was confirmed by the local pathology department (Table 2). Samples originated from different stages and tissues, including a primary tumor of the femur (C‐ES‐Y), a progression of pulmonary metastases (C‐ES‐I), ascites at relapse (C‐ES‐M), and second relapse affecting the humerus (C‐ES‐P). A comprehensive description of clinical details is provided in Table 2. The cell lines were designated according to the Cooperative Ewing Sarcoma Study (CESS) of the University Hospital Essen and deposited in the cell bank of the German Collection of Microorganisms and Cell Cultures (DSMZ) as a distributor for worldwide scientific use.

**TABLE 2:** Background information.

	C‐ES‐I	C‐ES‐M	C‐ES‐P	C‐ES‐Y
Sex	Female	Male	Female	Female
Age	20	22	8	19
Diagnosis	Ewing sarcoma	Ewing sarcoma	Ewing sarcoma	Ewing sarcoma
Fusion	EWSR1::FLI1 (n.s.)	EWSR1::FLI1 (n.s.)	EWSR1::FLI1 (Type I)	EWSR1::FLI1 (n.s.)
Primary tumor	Pre‐/paravertebral	Retrovesical with neuroforaminal and os sacrum infiltration	Thoracic paravertebral	Femur
Metastasis	Primarily pulmonary	Pulmonary	Pulmonary	No evidence
Relapse/progress		Relapse	Relapse	
Tissue sample	Resection of pulmonary metastasis	Ascites (relapse)	Biopsy of humerus (second relapse)	Resection of primary tumor
Chemotherapeutic agents before sampling	Actinomycin, carboplatin, cyclophosphamide, doxorubicin, etoposide, ifosfamide, irinotecan, temozolomide, topotecan, vincristine	Actinomycin, doxorubicin, etoposide, ifosfamide, vincristine*	Actinomycin, carboplatin, cyclophosphamide, doxorubicin, etoposide, ifosfamide, irinotecan, temozolomide, topotecan, vincristine	Cyclophosphamide, denosumab, doxorubicin, etoposide, ifosfamide, vincristine
Radiotherapy before sampling	Pulmonary	Region of primary tumor	Primary tumor and pulmonary metastasis	
Outcome	DOD	DOD	DOD	DOD

*Note*: Patient and treatment data up to the time point of tissue sampling. Chemotherapeutic agents are shown in alphabetic, not chronologic order. The asterisk (*) marks that the shown chemotherapeutic agents relate only to the relapsed case (9 years apart from primary disease).

Abbreviations: DOD, died of disease; n.s., not specified.

### 3.2 Genetic stability, authenticity, and immortality

The generation of STR profiles of the early and late passages of the cell lines did not show any similarities with each other or within the international STR reference database of cell lines,^23^ which approached or even exceeded the 80% similarity rule of an unrelated cell line.^24^ The authenticity and correct classification of the C‐ES cell lines could be achieved by congruence with the STR profile of the patients or biopsies. Even after 6 months of continuous cultivation, the heterozygosity of the STR profiles at the measured STR sites could be confirmed without modification. Interestingly, the STR profiles of STA‐ET‐1 varied between early and late passage due to allelic drifting (Table S2). A final investigation for a possible microsatellite instability (MSI) with the mononucleotide markers BAT25/26 proved the C‐ES cell lines as microsatellite‐stable (MSS), while shortening of both BAT markers indicated MSI in the case of STA‐ET‐1 (Figure S1), in line with previous observations.^25^

Since the C‐ES cell cultures exceeded the Hayflick limit with more than 80 passages, a proof of the immortality status was undertaken, and the cell lines were subjected to the recently published f‐TRAP assay for measuring induced telomerase activity (TA).^11^ In this specific assay, TA elongates a fluorescent oligonucleotide bait only when catalytically active telomerase is present and generates a ladder of elongated PCR products. The f‐TRAP examination of the C‐ES series and for STA‐ET‐1 showed in all cell lines TA in the same order of magnitude, which confirms the immortalization of EwS cells by activation of TA (Figure S2).

### 3.3 Morphology and proliferation rate

**FIGURE 1:**
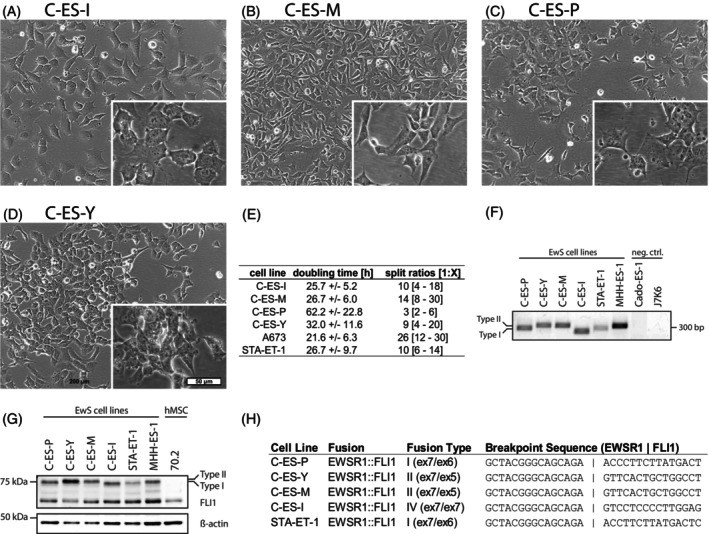
Morphology and basic characteristics. (A–D) Microscopy pictures of the Ewing sarcoma cell lines. Overlapping images in the lower right corners originate from separate images with 2× zoom. Images share the same scale bars depicted in (D). (E) Table of doubling time results from cell counting with trypan blue exclusion method and splitting ratios used twice weekly with different cell lines. The median split ration is shown with the interval of lowest and highest split ratios used in square brackets. (F) Agarose gel of EWSR1::FLI1 breakpoint region amplicons. STA‐ET‐1 and MHH‐ES1 served as positive controls for fusion types I and II, respectively. Cado‐ES1 (EWSR1‐ERG fusion) and J7K6 (hMSC) served as negative controls. Note that the amplicon size of C‐ES‐I is smaller than the expected size for fusion type I or II. (G) FLI1 western blot analysis showing a second and higher molecular weight band, indicating the EWSR1::FLI1 fusion protein. SK‐N‐MC and STA‐ET‐1 served as positive controls for EWSR‐FLI fusion type I. The hMSC 70.2 served as negative control. (H) Table of details on fusion types with respective breakpoint sequences obtained from Sanger sequencing.

Morphologically, the cell lines present rather diverse. C‐ES‐I shows the largest and most widespread cell bodies (Figure 1A). C‐ES‐M cells are small, and their shape is reminiscent of fibroblasts (Figure 1B). C‐ES‐P presents with rather large cell bodies and several needle‐like protrusions (Figure 1C). C‐ES‐Y cells differ in their size and show few protrusions (Figure 1D). C‐ES‐I, C‐ES‐M, C‐ES‐P, and C‐ES‐Y grow in adherent monolayers (Figure 1A–D). C‐ES‐Y tends to form loosely adherent spheroids under low cell density or suboptimal coating conditions.

The proliferation rates of the cell lines were assessed by counting viable cells after trypan blue counterstaining. A‐673 served as a control as it is a widely used EwS cell line with a population doubling time (PDT) of 24–25 h.^26^, ^27^ In our experiment, a doubling time of 22 h was determined for A‐673 (Figure 1E). Furthermore, we included the cell line STA‐ET‐1, which we always kept in culture in parallel with our newly generated cell lines in all subsequent experiments. The inclusion of STA‐ET‐1 served as a control as it is a commonly used EwS cell line.^28^ For STA‐ET‐1, we determined a doubling time of about 27 h. For C‐ES‐I and C‐ES‐M, similar doubling times of about 26–27 h were determined. C‐ES‐Y showed a prolonged doubling time of about 32 h. C‐ES‐P exhibited the lowest proliferation rate with a PDT of 62 h. The doubling times largely corresponded to the splitting ratios (Figure 1E).

### 3.4 Confirmation of *EWSR1::FLI1* translocation

The presence of the *EWSR1::FLI1* fusion transcript (Figure 1F) and corresponding fusion protein (Figure 1G), indicated by the characteristic second band at about 68 kDa,^29^ verified the identity of the four newly established EwS cell lines. The cell lines exhibit three different sizes of *EWSR1::FLI1*‐specific PCR products and EWSR1::FLI1 proteins, respectively (Figure 1F,G). The PCR products were subjected to Sanger sequencing to check the presumably different exon/exon transitions between the genes of *EWSR1* and *FLI1*. The analysis revealed an *EWSR1::FLI1* exon 7/5 fusion in the cell lines C‐ES‐M and C‐ES‐Y, an exon 7/6 fusion in C‐ES‐P, and an exon 7/7 fusion in C‐ES‐I (Figure 1H, Supplementary SangerSeq Data).

The analysis revealed fusion type I with an exon 7/6 transition (C‐ES‐P), two fusion types II with an exon 7/5 transition (C‐ES‐Y, C‐ES‐M), and fusion type IV with an exon 7/7 transition (C‐ES‐I). To our knowledge, fusion type IV has not yet been described in an established, continuous EwS cell line (Figure 1H).

### 3.5 The EwS cells show typical chromosomal aberrations

**FIGURE 2:**
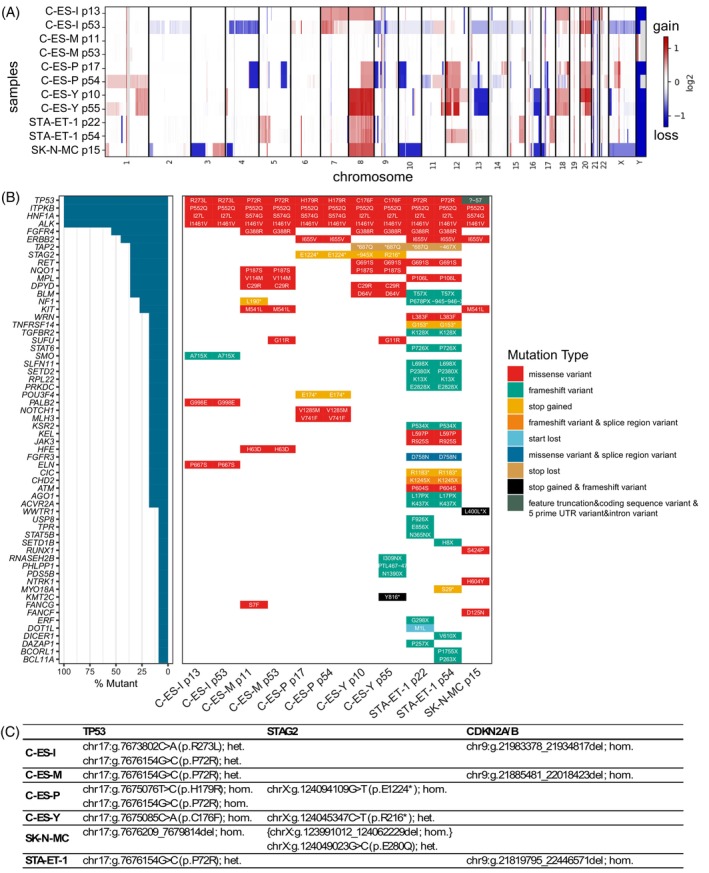
Genetic profiles. (A) Summarizing heatmap of CNV analysis. Red indicates gain and blue loss of chromosomal regions compared to standard human genome (GRCh38). The reference chromosome set was adjusted regarding the patient's sex. (B) Waterfall plot of genes with (likely) pathogenic alterations and alterations of controversial pathogenicity ordered by the frequency of observation in the C‐ES series. The box color depicts the type of mutation. Boxes are labeled with amino acid changes or the amino acid position at the site of a frameshift, respectively. (C) Tabular overview of genetic aberrations in TP53, STAG2, CDKN2A and CDKN2B in the analyzed EwS cell lines. het., heterozygous; hom., homozygous; {}, potentially subclones present.

The analysis of CNV at early and later time points during in vitro cultivation aimed to determine the chromosomal integrity. CNV analysis revealed several chromosomal aberrations in the newly generated EwS cell lines. The most frequent aberration in the C‐ES cell lines was an amplification of chromosome 8 (Figure 2A), which is the most common chromosomal gain in EwS.^5^, ^30^, ^31^, ^32^ Two cell lines had remarkable features in comparison to the others. Firstly, C‐ES‐M showed only a few CNVs and no large alterations (see also Figure S3). Secondly, C‐ES‐I showed significant changes in its chromosome set over time, while the chromosomal aberrations of the other cell lines remained rather stable (see also Figure S3).

In the cell lines C‐ES‐I and C‐ES‐M, CNV analysis further revealed large homozygous deletions comprising the whole gene locus of *CDKN2A/B* (Figure S3). C‐ES‐P lost one copy of the chromosomal region comprising *CDKN2A/B* (Figure S3). In addition, our results confirmed the homozygous deletion of *CDKN2A/B* in STA‐ET‐1 cells (Figure S3).^28^

Only one *STAG2* copy was found in C‐ES‐I, C‐ES‐M, and STA‐ET‐1 cells (Figure S3). The commercially available, established EwS cell line SK‐N‐MC presented with a homozygous deletion within the *STAG2* gene locus comprising the exons 2–17 (Figure S3). Due to the deletion of exon 3 containing the start codon, it can be considered a complete loss, which explains the previously reported lack of expression.^33^

### 3.6 Identification of additional mutations common to EwS

*TP53* was the most frequent hit in the variant analysis of the newly generated EwS cell lines (Figure 2B). The single nucleotide variants (SNVs) of *TP53* have been confirmed by Sanger sequencing (Supplementary Sanger Seq Data). In detail, the *TP53* mutations led to the following amino acid exchanges: C‐ES‐I: R273L (heterozygous) and P72R (heterozygous); C‐ES‐M: P72R (heterozygous); C‐ES‐P: H179R (homozygous) and P72R (homozygous); C‐ES‐Y: C176F (homozygous); STA‐ET‐1: P72R (heterozygous) (Figure 2C). The variants C176F, H179R and R273L are located in the DNA‐binding domain (DBD) of TP53.^34^ They have no transactivation activity^35^, ^36^, ^37^, ^38^ and increase migration and invasion capacities in vitro.^39^, ^40^ The P72R variant is located in the proline‐rich domain (PRD) of TP53.^34^ It is considered a common polymorphism and leads to a functional protein with respect to transcriptional activity, apoptosis and cell proliferation.^41^ Thus, it is often referred to as benign. However, P72R can act as a modifier of DBD mutant TP53 variants.^42^ The combination of P72R with DBD mutants led to increased migration and invasion in vitro, increased tumor volumes and metastasis in vivo, and can induce resistance to chemotherapeutic agents in vitro in some cases.^43^, ^44^, ^45^ SNV and CNV analyses also detected the known homozygous large deletion surrounding the start codon of *TP53* in SK‐N‐MC, which can be considered a complete loss (Figures 2B,C, S4).

Besides *TP53* alterations, coding variants of *STAG2* frequently occur in EwS.^5^, ^33^ In our analysis, we found two truncating mutations of *STAG2*. C‐ES‐P cells presented with a stop codon gain at protein position E1224, C‐ES‐Y cells at position R216 (Figure 2B). The R216* mutation truncates the bulk of the protein and is considered a complete loss. The loss of STAG2 can lead to aneuploidy, increased migratory capacity, and chemo‐resistance in vitro.^46^, ^47^ The mutation E1224* truncates the last nine amino acids of the STAG2 protein. To our knowledge, there is no information on its significance available.

The cell lines presented with variants in other genes (*ATM*, *ERBB2/HER2*, *FGFR3*, *FGFR4*, *NF1*, *NOTCH1*, *SMO*, and *SETD2*), commonly found in EwS patient samples.^48^

The other top candidate variant genes found in all cell lines were *ALK*, *HNF1A*, and *ITPKB*. However, these variants are poorly annotated but are likely to be benign according to ClinVar^49^ (https://www.ncbi.nlm.nih.gov/clinvar/).

In our tumor‐only analyses in the Varlociraptor pipeline, some candidate variants were only detected in early or late passage, respectively (Figure 2B). Analyzing these hits further, we found them in varying proportions in both early and late passage, indicating that the variants were already present in subclones of the original tumor, with the subclonal populations varying over time. When running the analysis pipeline again for direct comparison of late versus early passage (by annotating the early passage as normal control and the late passage as tumor sample), there were no hits (data not shown). Therefore, we assume that there was no introduction of cultivation‐induced genetic variants over the period of observation, at least none that had outperformed the existing subclonal populations.

### 3.7 Identification of potential factors that have influenced transcriptional variation within our series

Transcriptomic profiles were assessed by microarray. A principal component analysis (PCA) was performed to address differences in gene expression profiles between cell lines as well as between early and late passage of the same cell line. The commercially available and widely used EwS cell lines STA‐ET‐1 and SK‐N‐MC served as controls.

**FIGURE 3:**
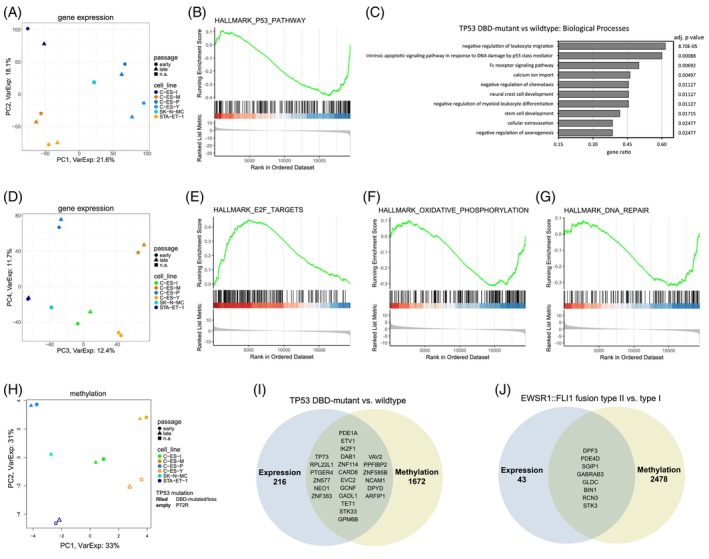
Transcriptomics. (A) PCA plot of the first and second PC of the microarray data. TP53 DBD mutant cells are colored in shades of blue, TP53 DBD wildtype cells are colored in shades of orange. (B) GSEA plot of the enriched hallmark “P53 pathway” in the comparison of TP53 DBD mutant versus wildtype cells. (C) Top GO terms (biological processes) by gene ratio from ORA of the comparison of TP53 DBD mutant versus wildtype cells. (D) PCA plot of the third and fourth PC of the microarray data. Cells are colored by EWSR1::FLI1 fusion type. Blue shades indicate exon 7–exon 6 (type I), orange shades exon 7–exon 5 (type II) and green exon 7‐exon 7 (type IV). (E) GSEA plot of the enriched hallmark “G2M checkpoint” in the comparison of cells with type II versus type I fusion. (F) Bar plots on the expression of the two most upregulated and the two most downregulated DEGs in the comparison of type II versus type I fusion. (G) GSEA plot of the enriched hallmark “KRAS signaling up” in the comparison of C‐ES‐M and C‐ES‐P cells versus the other cells. (H) PCA plot of the first and second PC of the methylation data. Cells with fusion type I are colored in shades of blue, cells with fusion type II are colored in shades of orange, and C‐ES‐I (fusion type IV) is colored in green. TP53 DBD mutant cells are depicted with filled symbols, TP53 DBD wildtype cells with empty symbols. (I) Top GO terms by gene ratio in the comparison of TP53 DBD mutant versus wildtype. (J) Venn diagram of the overlap between DEGs and DMGs in the comparison of type II versus type I fusion.

The first and second principal component (PC) together explained 39.7% of the variance and grouped the cell lines roughly according to the mutational state of the *TP53* DBD (Figure 3A). In gene set enrichment analysis (GSEA) comparing *TP53* DBD‐mutant (including complete *TP53* loss) with DBD‐wildtype cells, we found significant enrichment of the hallmarks MYC targets V1, E2F targets, oxidative phosphorylation, G2M checkpoint, MTORC1 signaling, MYC targets V2, estrogen response late, P53 pathway (Figure 3B), epithelial‐mesenchymal transition, PI3K‐AKT‐MTOR signaling, adipogenesis, mitotic spindle, unfolded protein response, and fatty acid metabolism. We found 240 differentially expressed genes (DEGs) in cells with mutations in the DBD of *TP53* compared to cells with wildtype DBD. Of these, 76 were upregulated and 164 downregulated. The extended DEGs (threshold: adjusted *p* value <.1) were used for over‐representation analysis (ORA) to search for enriched GO biological processes (Figure 3C). Both GSEA and ORA showed significant enrichment of *TP53*‐related hallmarks/terms (Figure 3B.C), indicating that part of the variance observed between the cell lines could potentially be explained by the mutational state of the DBD of *TP53*.

The third PC explained 12.4% of the variance in gene expression and grouped the cells according to their *EWSR1::FLI1* fusion type (Figure 3D). In GSEA, comparing cells with type II versus type I fusion, we found enrichment of the hallmarks G2M checkpoint, E2F targets (Figure 3E), mitotic spindle, and interferon gamma response. In this comparison, 42 genes were significantly upregulated and 9 downregulated. In ORA, there were no significantly enriched terms (data not shown). The type IV fusion cells C‐ES‐I were located between fusion type I and type II cells on the PC3 axis (Figure 3D).

PC4 explained 11.7% of the variance in gene expression and separated C‐ES‐M and C‐ES‐P from the rest of the cells (Figure 3D). GSEA found significant enrichment of the hallmarks MYC targets V2, KRAS signaling up, UV response up, UV response down, oxidative phosphorylation (Figure 3F), DNA repair (Figure 3G), epithelial‐to‐mesenchymal transition, reactive oxygen species pathway, and estrogen response early. These results indicate that C‐ES‐M and C‐ES‐P cells might have an altered DNA damage response and energy metabolism compared to the other cell lines (Figure 3F,G).

Furthermore, we analyzed whether there were changes in gene expression in the cell lines over time. Rather short distances between early and late passage of the same cell line in the first four principal components, which together explain 63.8% of the variance in gene expression, indicated that the delta in culturing period is not a major factor (Figure 3A,D). Furthermore, there was no commonality in the direction of the variance between early and late passage. Dividing the samples into two groups, early and late passage, no DEGs were identified (data not shown).

PCA on methylation sequencing data revealed similar patterns as for the transcriptome analysis. PC1 explained 33% of the variance, sorting the cell lines by their fusion type (Figure 3H). PC2 covers 30% of the variance and orders the cells by *TP53* mutational status (Figure 3H). Comparing *TP53* DBD mutant and *TP53* wildtype cells, 1696 regions overlapping with gene loci were differentially methylated. Of these, 24 genes were differentially expressed (Figure 3I). In the comparison of type II and type I fusion cells, 2486 regions overlapping gene loci were differentially methylated. Of these, eight genes were differentially expressed (Figure 3J).

### 3.8 The cell lines present with different drug sensitivity profiles

**FIGURE 4:**
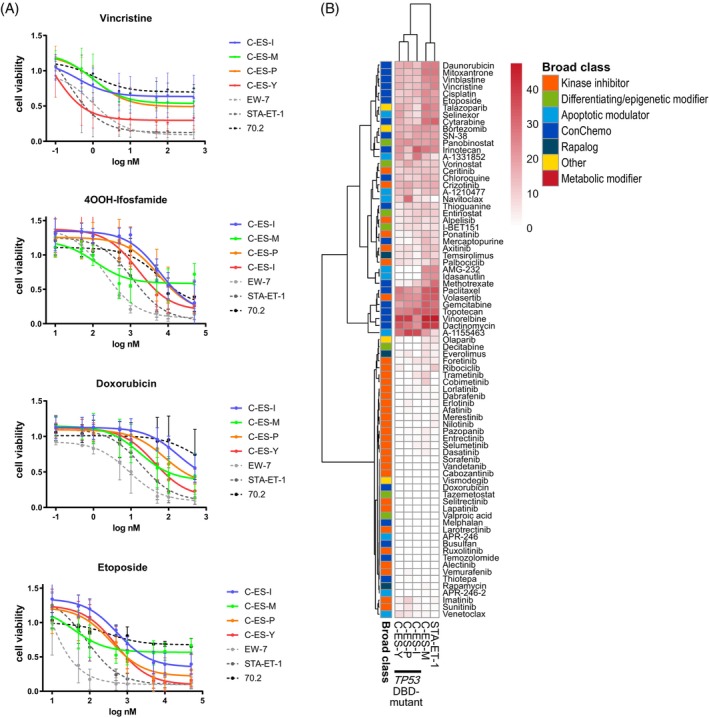
Drug screening. (A) Dose–response curves of the chemotherapeutics vincristine, 4OOH‐ifosfamide, doxorubicin and etoposide in cell viability assays. EwS cell lines EW‐7 and STA‐ET‐1 and hMSC 70.2 are depicted as dotted lines and serve as references. (B) Unsupervised hierarchical clustering of drug sensitivity scores. EwS cell line STA‐ET‐1 serves as a reference.

To gain insights into the drug responsiveness of the EwS cell lines, we performed cell viability assays with the standard chemotherapeutics vincristine, doxorubicin, etoposide, and an active form of ifosfamide. The new EwS cell lines showed higher resistance than the commercially available cell lines EW‐7 and STA‐ET‐1 (Figure 4A).

In addition, we performed drug sensitivity profiling (DSP) with a drug panel comprising 80 clinically relevant agents as part of the pediatric precision oncology program INFORM.^21^, ^22^ The DSP is illustrated by unsupervised hierarchical clustering based on the drug sensitivity scores (DSS_asym).^22^ The clustering groups cell lines in accordance with their drug efficacy and reflects the *TP53* mutation status (Figure 4B). The *TP53* DBD‐mutant cells showed decreased sensitivity to the MDM2 inhibitors idasanutlin and AMG‐232 and the conventional chemotherapeutics etoposide and vincristine. In contrast, the sensitivity to the BCL‐XL inhibitor A‐1155463 was increased in *TP53* DBD‐mutant cells. Interestingly, C‐ES‐P is less sensitive to its derivative A1331852 and is sensitive to the BCL‐2 inhibitor Navitoclax (Figure 4B).

The cell lines of the C‐ES series showed resistance to the chemotherapeutics doxorubicin and temozolomide, while showing the highest sensitivity to vinorelbine, except for CESI (Figure 4B). C‐ES‐I presented as most sensitive to the chemotherapeutics irinotecan and topotecan as well as the apoptotic modulators A‐1331852 and A‐1155463 (Figure 4B).

## 4 DISCUSSION

We validated the authenticity, immortality, and stability of the here presented, newly established EwS cell lines CESI, CESM, CESP, and C‐ESY. By comparing the STR profiles between donor material and the cell lines, authenticity was confirmed. Previously published data on the immortalization of sarcoma cell lines suggest that a predominant telomerase activation without alternative telomere elongation may be characteristic for sarcomas with specific chromosomal translocations such as EwS.^50^ Evidence of telomerase activation in all four cases seems to support the assumption that reprogramming occurs through alterations of the epigenome and methylome, most likely directly mediated by the chimeric transcription factor *EWSR1::FLI1*. Recently, it was shown that *EWSR1::FLI1*‐mediated reprogramming of 3D chromatin structures can promote an altered transcriptional state across different EwS cell lines.^51^

For in‐depth characterization of the cell lines, we collected data for the analysis of the genome, methylome, and transcriptome. The cell lines presented with typical chromosomal aberrations and mutations observed in EwS, for example, the gain of chromosome 8, the loss of *CDKN2A*, as well as *STAG2* and *TP53* mutations.^5^, ^30^, ^31^ Although other mutations besides the translocation are rare in Ewing sarcoma, *STAG2*, *CDKN2A*, and *TP53* mutations are the most common ones, with about 17%, 12%, and 7% occurrence rates in patients, respectively. In cell lines, however, the occurrence of additional mutations is much higher.^5^ There are multiple possible reasons for the higher occurrence rate of mutations in cell lines. The number of sampling opportunities of tumors with mutations that lead to more aggressive phenotypes might be higher, for example, because of higher frequencies of relapse, metastasis, and refractory disease. Furthermore, EwS cells with additional mutations might grow more easily under culture conditions. Another explanation is that mutated subclones are selected early during cell line generation because of their enhanced proliferative capacities.^52^

While types I and II are the most frequent *EWSR1::FLI1* fusions, other fusion types are rarely observed in EwS patients.^53^ Thus, the availability of EwS cell lines with other *EWSR1::FLI1* fusions despite types I and II is limited. Strikingly, C‐ES‐I is the first EwS cell line described with an *EWSR1::FLI1* type IV fusion and represents an important extension of the current spectrum of EwS cell lines.

In transcriptome analysis of the C‐ES series, the *EWSR1::FLI1* fusion type and the mutational status of the *TP53* DBD were identified to be potential factors that influenced the variance in gene expression and DNA methylation. However, in larger‐scale studies, these factors have not been identified to be playing a major role^15^, ^54^ and the *EWSR1::FLI1* fusion type alone does not seem to have clinical significance in current therapies.^4^ Still, tumor heterogeneity feeds from multiple factors and investigation of its mechanics may enable improvement in targeted therapy approaches in the future.

Since all the cell lines originated from patients with a dismal outcome, a drug sensitivity profiling was performed to investigate potential sensitivities. *TP53* DBD‐mutant cell lines presented with a different, less sensitive drug profile, resistance to MDM2 inhibitors, and sensitivity to BCL‐2 and BCL‐XL inhibitors. Yet, the best‐performing drug was the chemotherapeutic agent vinorelbine, presenting with rather high efficacies in both *TP53* DBD‐mutant and wildtype cell lines.

Our analyses also served to further characterize the commonly used EwS cell lines SK‐N‐MC and STAET‐1. We showed that SK‐N‐MC has a homozygous deletion in the *STAG2* gene locus leading to a complete loss. STAET‐1 showed a heterozygous deletion within *STAG2*.

In conclusion, we present four newly established EwS cell lines derived from patients in late childhood and early adulthood. The chimeric transcription factor *EWSR1::FLI1* as a driver of oncogenesis revealed known type I and II fusion variants and, for the first time for an in vitro EwS model, fusion variant IV. In the future, the C‐ES cell line series may play a role in analyzing reprogramming mechanisms by *EWSR1::FLI1* and exploring the involvement of transcriptional domains in EwS tumorigenesis.

## AUTHOR CONTRIBUTIONS

**Maximilian Kerkhoff:** Investigation; methodology; formal analysis; writing – original draft; visualization. **Christiane Schaefer:** Writing – original draft; investigation; methodology; formal analysis; visualization. **Wilhelm G. Dirks:** Investigation; writing – original draft; methodology; formal analysis; validation. **Dawid Krzeciesa:** Writing – review and editing; formal analysis; software; methodology; validation. **Maximilian Bretschneider:** Data curation; formal analysis. **Pauline R. Plaumann:** Investigation; methodology. **Wiebke K. Guder:** Resources; writing – review and editing. **Arne Streitbürger:** Resources; writing – review and editing; funding acquisition. **Sonja Herter:** Methodology; software; formal analysis; visualization. **Heike Peterziel:** Investigation; methodology; formal analysis; resources; writing – review and editing. **Ina Oehme:** Resources; supervision; writing – review and editing. **Felina Zahnow:** Methodology; investigation. **Thomas G. P. Grünewald:** Resources; supervision; writing – review and editing. **Uta Dirksen:** Resources; supervision; project administration; writing – review and editing; conceptualization; funding acquisition.

## FUNDING INFORMATION

This work was supported by the BILD hilft e.V. “Ein Herz für Kinder” (PÄ‐40605) to A. Streitbürger and U. Dirksen, the Reichert‐Alanod Foundation to M. Kerkhoff and U. Dirksen, and the Gert and Susanne Mayer Foundation (BMBF 01KD2207B [HEROES‐AYA]) to U. Dirksen and T.G.P. Grünewald. The laboratory of T.G.P. Grünewald is supported by the Barbara and Wilfried Mohr Foundation and is co‐funded by the European Union (ERC, CANCER‐HARAKIRI, 101122595). Views and opinions expressed are, however, those of the authors only and do not necessarily reflect those of the European Union or the European Research Council. Neither the European Union nor the granting authority can be held responsible for them.

## CONFLICT OF INTEREST STATEMENT

The authors have no conflicts of interest to declare.

## ETHICS STATEMENT

The biomaterial for this research project was provided by the Westdeutsche Biobank Essen (WBE, University Hospital Essen, University of Duisburg‐Essen, Essen, Germany) and approved by the institutional ethics committee of the University of Essen, Germany (12‐5279‐BO). This research work was conducted in accordance with the Declaration of Helsinki. All patients/their legal guardians gave written consent to biomaterial acquisition, processing, and analysis, including genomic characterization and sharing biomaterials with other researchers, limited to scientific purposes.

## Supporting information


**FIGURE S1:** BAT‐26 and BAT‐25 profiling for MSI detection. BAT‐26 reads are shown in blue, BAT‐25 reads in black. Colon cancer cell line HCT‐116 served as a positive control.
**FIGURE S2:** Detection of telomerase activity in EwS cell lines measured by f‐TRAP assay. EPGs of f‐TRAP PCR product analysis by CE are shown of 9 × 10^4^, 4.5 × 10^4^, or 9 × 10^3^ cells of C‐ES‐I, C‐ES‐M, C‐ES‐P, and C‐ES‐Y are shown, respectively. The numbers of telomeric repeats are not indicated but can be automatically evaluated for 6 bp GGGATT distance by adjusting the STR locus tags in the Genetic Analyzer software (Sciex, Darmstadt, Germany). Heat inactivation was performed by incubating the extension reactions for 10 min at 85°C temperature.
**FIGURE S3:** Copy number variations at specific gene regions. Logarithmic (log2) copy ratios of bins in the chromosomal region of interest, the yellow stripe highlights the respective gene locus. The passage of the sample is depicted in the lower left corner of the respective diagram. Human genome reference GRCh38/hg38.
**FIGURE S4:** TP53 deletion in SK‐N‐MC. Logarithmic (log2) copy ratios of bins in the chromosomal region of interest, the yellow stripe highlights the respective gene locus (left). Visualization of a deletion encompassing the start codon of TP53 in SK‐N‐MC cells by Integrative Genomics Viewer (IGV; right). Human genome reference GRCh38/hg38.

**TABLE S1:** Quality metrics of whole genome sequencing (WGS) and whole exome sequencing (WES). Low coverage WGS and WES data were mapped to the reference genome GRCh38.

**TABLE S2:** Search results of STR queries within the international STR reference database of human cell lines.^23^

## Data Availability

The data discussed in this publication have been deposited in NCBI's Gene Expression Omnibus^55^ and are accessible through GEO Series accession number GSE289118 (https://www.ncbi.nlm.nih.gov/geo/query/acc.cgi?acc=GSE289118). Further data that support the findings of this study are available from the corresponding author upon reasonable request. The C‐ES cell lines will be available to the scientific community at the biorepository German Collection of Microorganisms and Cell Cultures (https://www.dsmz.de/).
