# Supplementary material for: New cell lines expanding the diversity of Ewing sarcoma models
Source: Int J Cancer. 2025 Sep 26;158(3):642–55. doi: 10.1002/ijc.70172 (PMC12670343; doi:10.1002/ijc.70172)
Supplement: Supplementary file 1 — FIGURE S1: BAT‐26 and BAT‐25 profiling for MSI detection. BAT‐26 reads are shown in blue, BAT‐25 reads in black. Colon cancer cell line HCT‐116 served as a positive control. FIGURE S2: Detection of telomerase activity in EwS cell lines measured by f‐TRAP assay. EPGs of f‐TRAP PCR product analysis by CE are shown of 9 × 10^4^, 4.5 × 10^4^, or 9 × 10^3^ cells of C‐ES‐I, C‐ES‐M, C‐ES‐P, and C‐ES‐Y are shown, respectively. The numbers of telomeric repeats are not indicated but can be automatically evaluated for 6 bp GGGATT distance by adjusting the STR locus tags in the Genetic Analyzer software (Sciex, Darmstadt, Germany). Heat inactivation was performed by incubating the extension reactions for 10 min at 85°C temperature. FIGURE S3: Copy number variations at specific gene regions. Logarithmic (log2) copy ratios of bins in the chromosomal region of interest, the yellow stripe highlights the respective gene locus. The passage of the sample is depicted in the lower left corner of the respective diagram. Human genome reference GRCh38/hg38. FIGURE S4: TP53 deletion in SK‐N‐MC. Logarithmic (log2) copy ratios of bins in the chromosomal region of interest, the yellow stripe highlights the respective gene locus (left). Visualization of a deletion encompassing the start codon of TP53 in SK‐N‐MC cells by Integrative Genomics Viewer (IGV; right). Human genome reference GRCh38/hg38. [file IJC-158-642-s003.pdf]

International Journal of Cancer

New Cell lines Expanding the Diversity of Ewing Sarcoma Models

Maximilian Kerkhoff, Christiane Schaefer, Wilhelm G. Dirks, Dawid Krzeciesa, Maximilian Bretschneider, Pauline R. Plaumann, Wiebke K. Guder, Arne Streitbürger, Sonja Herter, Heike Peterziel, Ina Oehme, Felina Zahnow, Thomas G. P. Grünewald & Uta Dirksen

Supplementary Data

Table of Contents

Supplementary Figure 1: BAT-26 and BAT-25 profiling for MSI detection

Supplementary Figure 2: Detection of telomerase activity in EwS cell lines measured by f-TRAP assay

Supplementary Figure 3: Copy number variations at specific gene regions

Supplementary Figure 4: TP53 deletion in SK-N-MC

Supplementary Sanger Sequencing Data

Data can be found in separate files for

Supplementary Table 1: Quality Control NGS

Supplementary Table 2: STR profiles

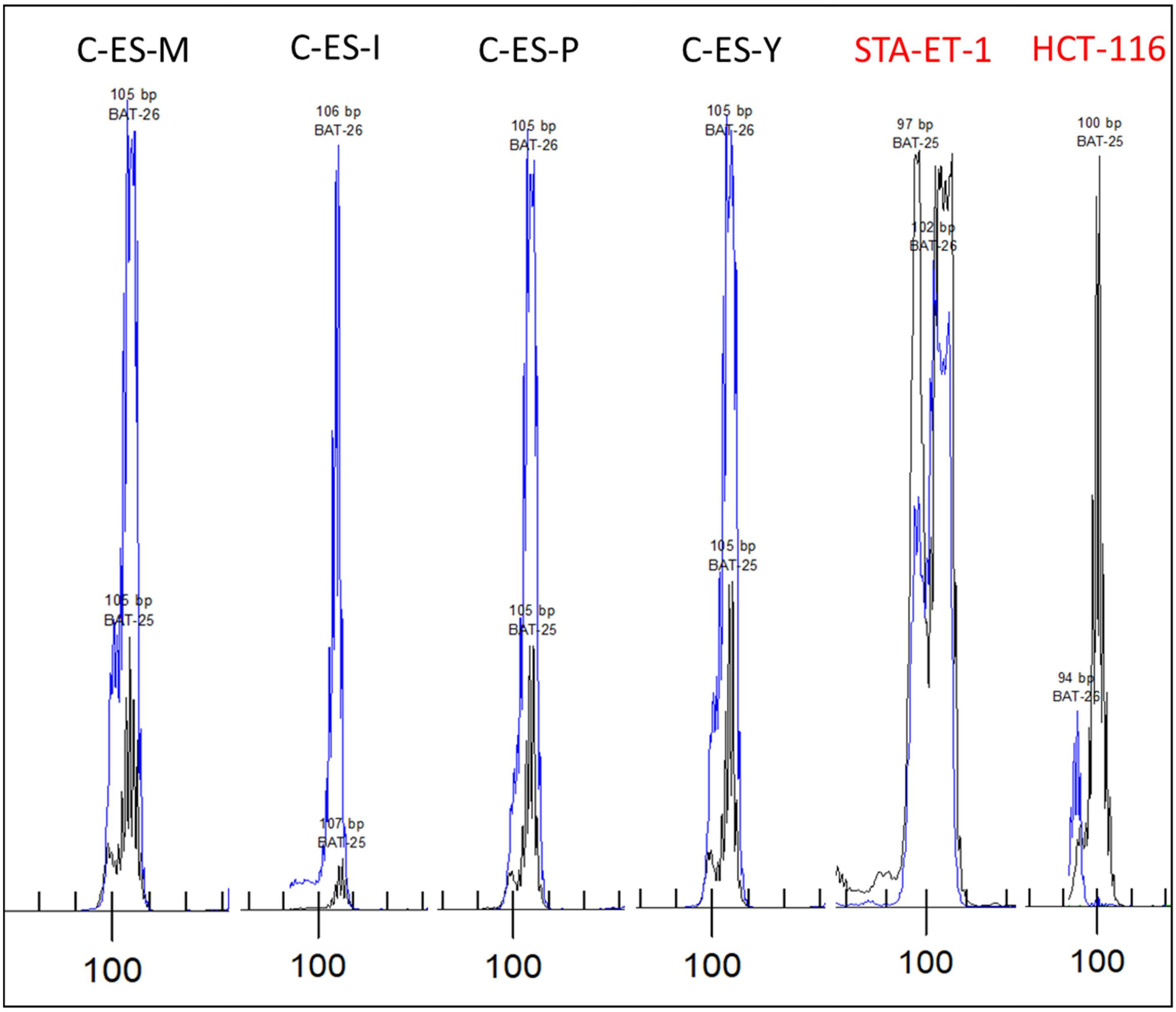

Supplementary Figure 1. BAT-26 and BAT-25 profiling for MSI detection. BAT-26 reads are shown in blue, BAT-25 reads in black. Colon cancer cell line HCT-116 served as a positive control.

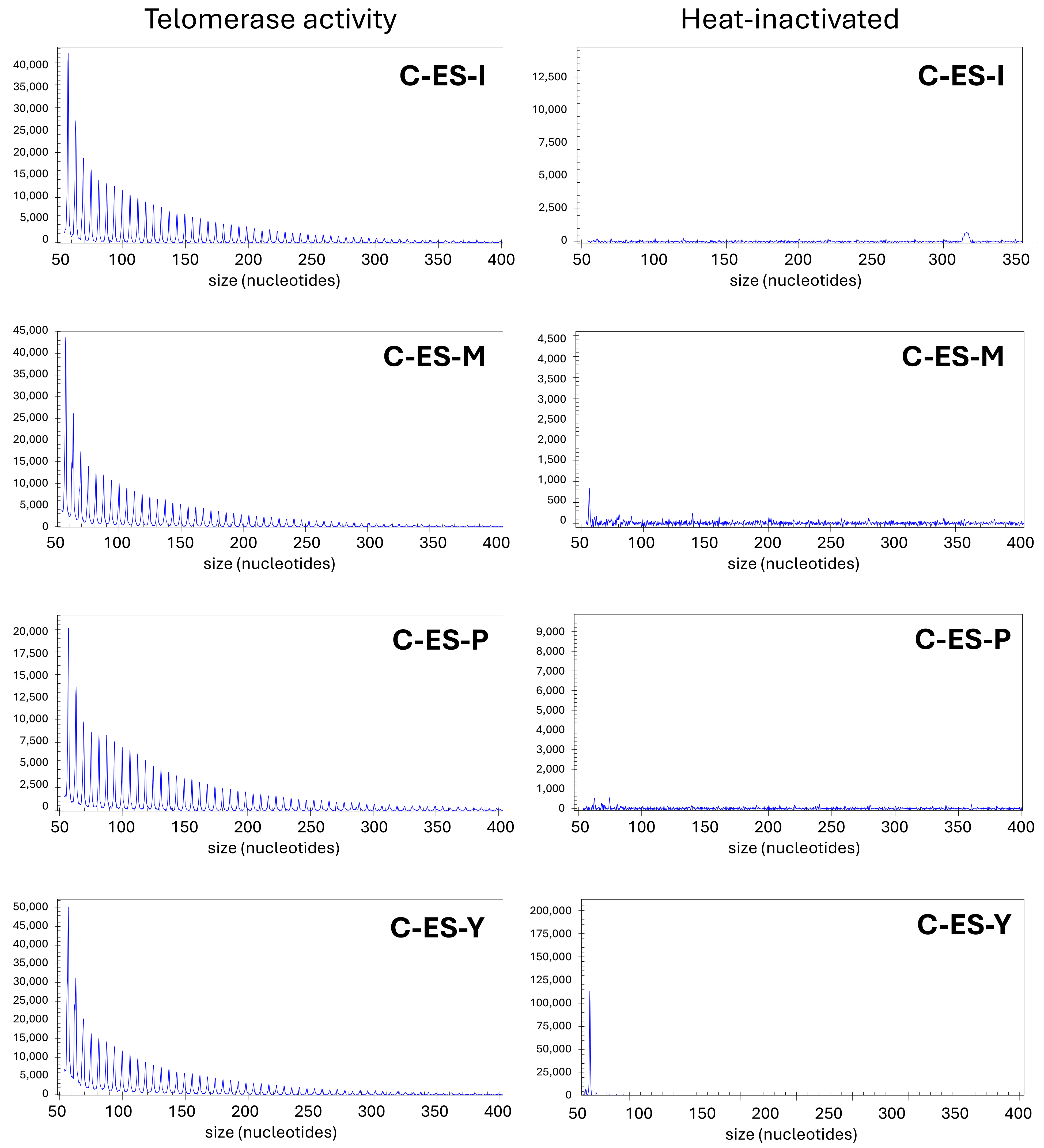

Supplementary Figure 2. Detection of telomerase activity in EWS cell lines measured by f-TRAP assay. EPGs of f-TRAP PCR product analysis by CE are shown of $9x10^4$, 4,5 x 10^4 or $9x10^3$ cells of C-ES-I, C-ES-M, C-ES-P and C-ES-Y are shown, respectively. The numbers of telomeric repeats are not indicated but can be automatically evaluated for 6 bp GGGATT distance by adjusting the STR locus tags in the Genetic Analyzer software (Sciex, Darmstadt, Germany). Heat inactivation was performed by incubating the extension reactions for 10 minutes at 85° Celsius temperature.

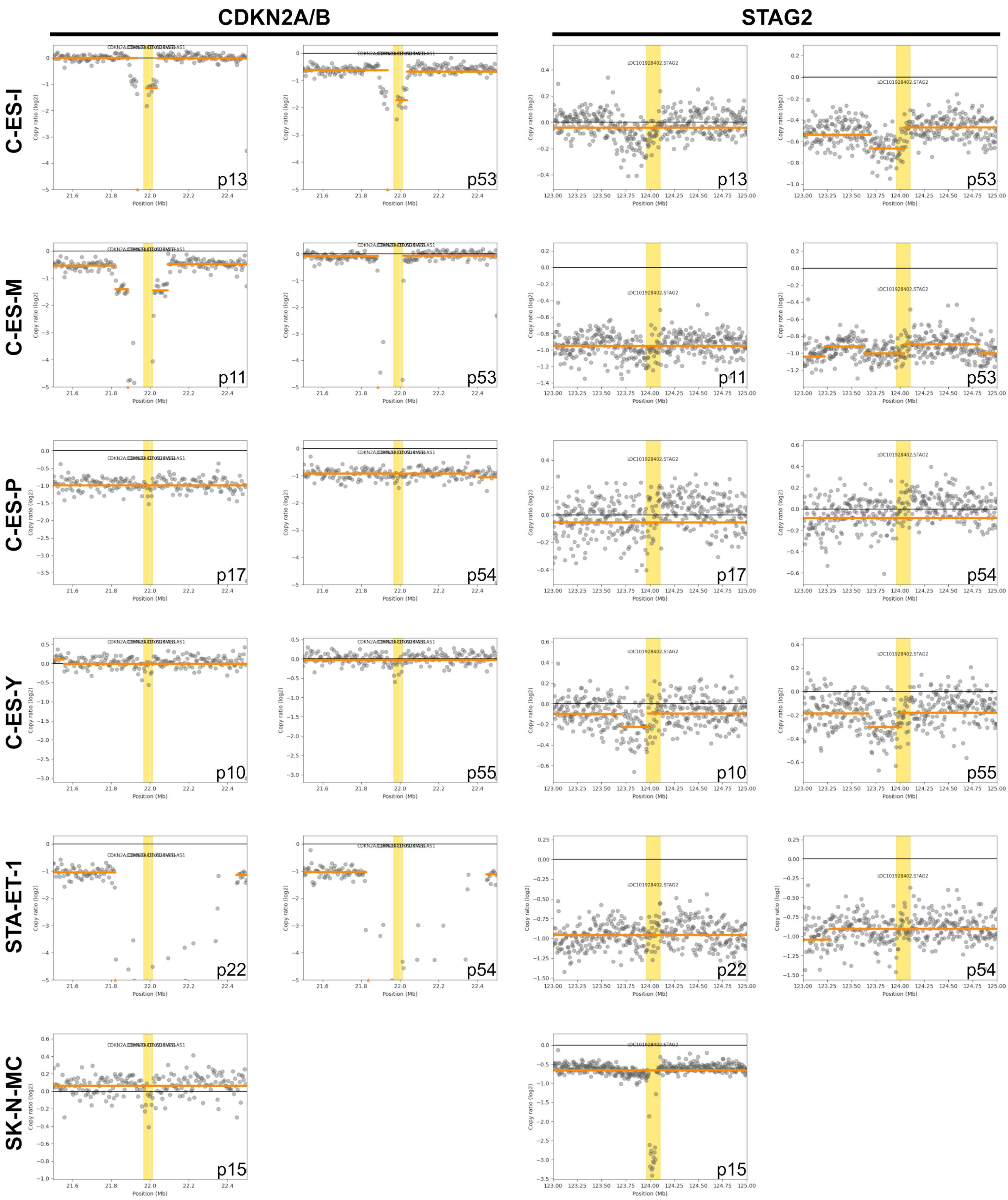

Supplementary Figure 3. Copy number variations at specific gene regions. Logarithmic (log2) copy ratios of bins in the chromosomal region of interest, the yellow stripe highlights the respective gene locus. The passage of the sample is depicted in the lower left corner of the respective diagram. Human genome reference GRCh38/hg38.

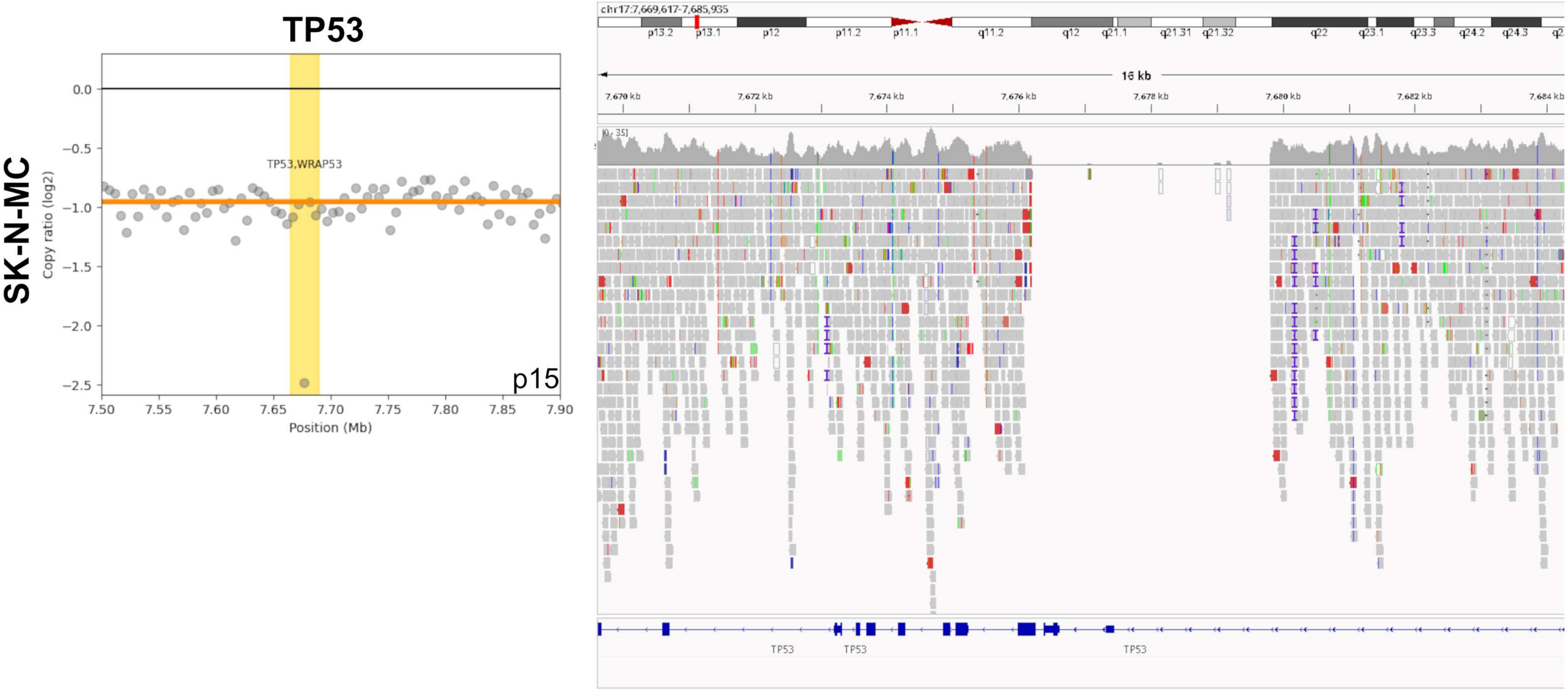

Supplementary Figure 4. TP53 deletion in SK-N-MC. Logarithmic (log2) copy ratios of bins in the chromosomal region of interest, the yellow stripe highlights the respective gene locus (left). Visualization of a deletion encompassing the start codon of TP53 in SK-N-MC cells by Integrative Genomics Viewer (IGV; right). Human genome reference GRCh38/hg38.

C-ES-I

```
Alignment of Sequence_1:  [EWSR1-FLI1 Type IV CDS.xdna]
with  Sequence_2: [C-ES-I EWSR1-FLI1_seq_rev.xdna]

              P  P  Q  T  G  S  Y  S  Q  A  P  S  Q  Y  S  Q  Q  S  S  S
Seq_1  721    CCACCCCAAACTGGATCCTACAGCCAAGCTCCAAGTCAATATAGCCAACAGAGCAGCAGC  780
                                                                    ||||||
Seq_2  109    ------------------------------------------------------AGCAGC  104
                                                                    S  S

                    EWSR1    FLI1
              Y  G  Q  Q  S  P  P  L  G  G  A  Q  T  I  S  K  N  T  E  Q
Seq_1  781    TACGGGCAGCAGAGTCCTCCCCTTGGAGGGGCACAAACGATCAGTAAGAATACAGAGCAA  840
              ||||||||||||||||||||||||||||||||||||||||||||||||||||||||||||
Seq_2  103    TACGGGCAGCAGAGTCCTCCCCTTGGAGGGGCACAAACGATCAGTAAGAATACAGAGCAA  44
              Y  G  Q  Q  S  P  P  L  G  G  A  Q  T  I  S  K  N  T  E  Q

              R  P  Q  P  D  P  Y  Q  I  L  G  P  T  S  S  R  L  A  N  P
Seq_1  841    CGGCCCCAGCCAGATCCGTATCAGATCCTGGGCCCGACCAGCAGTCGCCTAGCCAACCCT  900
              |||||||||||||||||||||||||||||||||||||||||||
Seq_2  43     CGGCCCCAGCCAGATCCGTATCAGATCCTGGGCCCGACCAGCA-----------------  1
              R  P  Q  P  D  P  Y  Q  I  L  G  P  T  S  X
```

C-ES-M

```
Alignment of Sequence_1:  [EWSR1-FLI1 Type II CDS.xdna]
with  Sequence_2: [C-ES-M EWSR1-FLI1_seq_rev.fasta]

              P  P  Q  T  G  S  Y  S  Q  A  P  S  Q  Y  S  Q  Q  S  S  S
Seq_1  721    CCACCCCAAACTGGATCCTACAGCCAAGCTCCAAGTCAATATAGCCAACAGAGCAGCAGC  780
                             |||||||||||||||||||||||||||||||||||||||||||||
Seq_2  280    ---------------TCCTACAGCCAAGCTCCAAGTCAATATAGCCAACAGAGCAGCAGC  236
                             S  Y  S  Q  A  P  S  Q  Y  S  Q  Q  S  S  S

                    EWSR1    FLI1
              Y  G  Q  Q  S  S  L  L  A  Y  N  T  T  S  H  T  D  Q  S  S
Seq_1  781    TACGGGCAGCAGAGTTCACTGCTGGCCTATAATACAACCTCCCACACCGACCAATCCTCA  840
              ||||||||||||||||||||||||||||||||||||||||||||||||||||||||||||
Seq_2  235    TACGGGCAGCAGAGTTCACTGCTGGCCTATAATACAACCTCCCACACCGACCAATCCTCA  176
              Y  G  Q  Q  S  S  L  L  A  Y  N  T  T  S  H  T  D  Q  S  S

              R  L  S  V  K  E  D  P  S  Y  D  S  V  R  R  G  A  W  G  N
Seq_1  841    CGATTGAGTGTCAAAGAAGACCCTTCTTATGACTCAGTCAGAAGAGGAGCTTGGGGCAAT  900
              ||||||||||||||||||||||||||||||||||||||||||||||||||||||||||||
Seq_2  175    CGATTGAGTGTCAAAGAAGACCCTTCTTATGACTCAGTCAGAAGAGGAGCTTGGGGCAAT  116
              R  L  S  V  K  E  D  P  S  Y  D  S  V  R  R  G  A  W  G  N
```

C-ES-P

```
Alignment of Sequence_1:  [EWSR1-FLI1 Type I CDS.xdna]
with  Sequence_2: [C-ES-P EWSR1-FLI1_seq_for.fasta]

             P  P  Q  T  G  S  Y  S  Q  A  P  S  Q  Y  S  Q  Q  S  S  S
Seq_1  721   CCACCCCAAACTGGATCCTACAGCCAAGCTCCAAGTCAATATAGCCAACAGAGCAGCAGC  780
                                                                        |
Seq_2  1     -----------------------------------------------------------C  1


                   EWSR1    FLI1
             Y  G  Q  Q  N  P  S  Y  D  S  V  R  R  G  A  W  G  N  N  M
Seq_1  781   TACGGGCAGCAGAACCCTTCTTATGACTCAGTCAGAAGAGGAGCTTGGGGCAATAACATG  840
             ||||||||||||||||||||||||||||||||||||||||||||||||||||||||||||
Seq_2  2     TACGGGCAGCAGAACCCTTCTTATGACTCAGTCAGAAGAGGAGCTTGGGGCAATAACATG  61
             Y  G  Q  Q  N  P  S  Y  D  S  V  R  R  G  A  W  G  N  N  M


             N  S  G  L  N  K  S  P  P  L  G  G  A  Q  T  I  S  K  N  T
Seq_1  841   AATTCTGGCCTCAACAAAAGTCCTCCCCTTGGAGGGGCACAAACGATCAGTAAGAATACA  900
             ||||||||||||||||||||||||||||||||||||||||||||||||||||||||||||
Seq_2  62    AATTCTGGCCTCAACAAAAGTCCTCCCCTTGGAGGGGCACAAACGATCAGTAAGAATACA  121
             N  S  G  L  N  K  S  P  P  L  G  G  A  Q  T  I  S  K  N  T
```

C-ES-Y

```
Alignment of Sequence_1:  [EWSR1-FLI1 Type II CDS.xdna]
with  Sequence_2: [C-ES-Y EWSR1-FLI1_seq_rev.fasta]

             P  P  Q  T  G  S  Y  S  Q  A  P  S  Q  Y  S  Q  Q  S  S  S
Seq_1  721   CCACCCCAAACTGGATCCTACAGCCAAGCTCCAAGTCAATATAGCCAACAGAGCAGCAGC  780
                            |||||||||||||||||||||||||||||||||||||||||||||
Seq_2  281   ---------------TCCTACAGCCAAGCTCCAAGTCAATATAGCCAACAGAGCAGCAGC  237
                            S  Y  S  Q  A  P  S  Q  Y  S  Q  Q  S  S  S

                   EWSR1    FLI1
             Y  G  Q  Q  S  S  L  L  A  Y  N  T  T  S  H  T  D  Q  S  S
Seq_1  781   TACGGGCAGCAGAGTTCACTGCTGGCCTATAATACAACCTCCCACACCGACCAATCCTCA  840
             ||||||||||||||||||||||||||||||||||||||||||||||||||||||||||||
Seq_2  236   TACGGGCAGCAGAGTTCACTGCTGGCCTATAATACAACCTCCCACACCGACCAATCCTCA  177
             Y  G  Q  Q  S  S  L  L  A  Y  N  T  T  S  H  T  D  Q  S  S


             R  L  S  V  K  E  D  P  S  Y  D  S  V  R  R  G  A  W  G  N
Seq_1  841   CGATTGAGTGTCAAAGAAGACCCTTCTTATGACTCAGTCAGAAGAGGAGCTTGGGGCAAT  900
             ||||||||||||||||||||||||||||||||||||||||||||||||||||||||||||
Seq_2  176   CGATTGAGTGTCAAAGAAGACCCTTCTTATGACTCAGTCAGAAGAGGAGCTTGGGGCAAT  117
             R  L  S  V  K  E  D  P  S  Y  D  S  V  R  R  G  A  W  G  N
```

STA-ET-1

Alignment of Sequence_1: [EWSR1-FLI1 Type I CDS]
with Sequence_2: [STA-ET-1 EWSR1-FLI1_seq_rev.fasta]

```
               P  P  Q  T  G  S  Y  S  Q  A  P  S  Q  Y  S  Q  Q  S  S  S
Seq_1  721     CCACCCCAAACTGGATCCTACAGCCAAGCTCCAAGTCAATATAGCCAACAGAGCAGCAGC  780
                               |||||||||||||||||||||||||||||||||||||||||||||
Seq_2  224     ---------------TCCTACAGCCAAGCTCCAAGTCAATATAGCCAACAGAGCAGCAGC  180
                              S  Y  S  Q  A  P  S  Q  Y  S  Q  Q  S  S  S

                      EWSR1    FLI1
               Y  G  Q  Q  N  P  S  Y  D  S  V  R  R  G  A  W  G  N  N  M
Seq_1  781     TACGGGCAGCAGAACCCTTCTTATGACTCAGTCAGAAGAGGAGCTTGGGGCAATAACATG  840
               |||||||||||||||||||||||||||||||||||||||||||| |||||||||||||||
Seq_2  179     TACGGGCAGCAGAACCCTTCTTATGACTCAGTCAGAAGAGGAGCATGGGGCAATAACATG  120
               Y  G  Q  Q  N  P  S  Y  D  S  V  R  R  G  A  W  G  N  N  M


               N  S  G  L  N  K  S  P  P  L  G  G  A  Q  T  I  S  K  N  T
Seq_1  841     AATTCTGGCCTCAACAAAAGTCCTCCCCTTGGAGGGGCACAAACGATCAGTAAGAATACA  900
               ||||||||||||||||||||||||||||||||||||||||||||||||||||||||||||
Seq_2  119     AATTCTGGCCTCAACAAAAGTCCTCCCCTTGGAGGGGCACAAACGATCAGTAAGAATACA  60
               N  S  G  L  N  K  S  P  P  L  G  G  A  Q  T  I  S  K  N  T
```

C-ES-I

Alignment of Sequence_1: [TP53 CDS.xdna] with Sequence_2: [Sanger Consensus]
Similarity : 1180/1234 (95,62 %)

```
                                        M  E  E  P  Q  S  D  P  S  V  E  P  P
Seq_1  1      ----------------------atggaggagccgcagtcagatcctagcgtcgagccccc  38
              ######################||||||||||||||||||||||||||||||||||||||
Seq_2  1      AACTGCCTTCCGGGTCACTGCCATGGAGGAGCCGCAGTCAGATCCTAGCGTCGAGCCCCC  60
               T  A  F  R  V  T  A  M  E  E  P  Q  S  D  P  S  V  E  P  P


               L  S  Q  E  T  F  S  D  L  W  K  L  L  P  E  N  N  V  L  S
Seq_1  39     tctgagtcaggaaacattttcagacctatggaaactacttcctgaaaacaacgttctgtc  98
              ||||||||||||||||||||||||||||||||||||||||||||||||||||||||||||
Seq_2  61     TCTGAGTCAGGAAACATTTTCAGACCTATGGAAACTACTTCCTGAAAACAACGTTCTGTC  120
               L  S  Q  E  T  F  S  D  L  W  K  L  L  P  E  N  N  V  L  S


               P  L  P  S  Q  A  M  D  D  L  M  L  S  P  D  D  I  E  Q  W
Seq_1  99     ccccttgccgtcccaagcaatggatgatttgatgctgtccccggacgatattgaacaatg  158
              ||||||||||||||||||||||||||||||||||||||||||||||||||||||||||||
Seq_2  121    CCCCTTGCCGTCCCAAGCAATGGATGATTTGATGCTGTCCCCGGACGATATTGAACAATG  180
               P  L  P  S  Q  A  M  D  D  L  M  L  S  P  D  D  I  E  Q  W

                                                                 P72R
               F  T  E  D  P  G  P  D  E  A  P  R  M  P  E  A  A  P  P  V
Seq_1  159    gttcactgaagacccaggtccagatgaagctcccagaatgccagaggctgctccccccgt  218
              ||||||||||||||||||||||||||||||||||||||||||||||||||||||||#|||
Seq_2  181    GTTCACTGAAGACCCAGGTCCAGATGAAGCTCCCAGAATGCCAGAGGCTGCTCCCCGCGT  240
               F  T  E  D  P  G  P  D  E  A  P  R  M  P  E  A  A  P  R  V


               A  P  A  P  A  A  P  T  P  A  A  P  A  P  A  P  S  W  P  L
Seq_1  219    ggcccctgcaccagcagctcctacaccggcggcccctgcaccagcccccctcctggcccct  278
              ||||||||||||||||||||||||||||||||||||||||||||||||||||||||||||
Seq_2  241    GGCCCCTGCACCAGCAGCTCCTACACCGGCGGCCCCTGCACCAGCCCCCTCCTGGCCCCT  300
               A  P  A  P  A  A  P  T  P  A  A  P  A  P  A  P  S  W  P  L


               S  S  S  V  P  S  Q  K  T  Y  Q  G  S  Y  G  F  R  L  G  F
Seq_1  279    gtcatcttctgtcccttcccagaaaacctaccagggcagctacggtttccgtctgggctt  338
              ||||||||||||||||||||||||||||||||||||||||||||||||||||||||||||
Seq_2  301    GTCATCTTCTGTCCCTTCCCAGAAAACCTACCAGGGCAGCTACGGTTTCCGTCTGGGCTT  360
               S  S  S  V  P  S  Q  K  T  Y  Q  G  S  Y  G  F  R  L  G  F


               L  H  S  G  T  A  K  S  V  T  C  T  Y  S  P  A  L  N  K  M
Seq_1  339    cttgcattctgggacagccaagtctgtgacttgcacgtactcccctgccctcaacaagat  398
              ||||||||||||||||||||||||||||||||||||||||||||||||||||||||||||
Seq_2  361    CTTGCATTCTGGGACAGCCAAGTCTGTGACTTGCACGTACTCCCCTGCCCTCAACAAGAT  420
               L  H  S  G  T  A  K  S  V  T  C  T  Y  S  P  A  L  N  K  M


               F  C  Q  L  A  K  T  C  P  V  Q  L  W  V  D  S  T  P  P  P
Seq_1  399    gttttgccaactggccaagacctgccctgtgcagctgtgggttgattccacacccccgcc  458
              ||||||||||||||||||||||||||||||||||||||||||||||||||||||||||||
Seq_2  421    GTTTTGCCAACTGGCCAAGACCTGCCCTGTGCAGCTGTGGGTTGATTCCACACCCCCGCC  480
               F  C  Q  L  A  K  T  C  P  V  Q  L  W  V  D  S  T  P  P  P


               G  T  R  V  R  A  M  A  I  Y  K  Q  S  Q  H  M  T  E  V  V
Seq_1  459    cggcacccgcgtccgcgccatggccatctacaagcagtcacagcacatgacggaggttgt  518
              ||||||||||||||||||||||||||||||||||||||||||||||||||||||||||||
Seq_2  481    CGGCACCCGCGTCCGCGCCATGGCCATCTACAAGCAGTCACAGCACATGACGGAGGTTGT  540
               G  T  R  V  R  A  M  A  I  Y  K  Q  S  Q  H  M  T  E  V  V
```

```
                R  R  C  P  H  H  E  R  C  S  D  S  D  G  L  A  P  P  Q  H
Seq_1   519   gaggcgctgcccccaccatgagcgctgctcagatagcgatggtctggcccctcctcagca   578
              |::|||||||||||||||||||||||||||||||||||||||||||||||||||||||||
Seq_2   541   GRRGCGCTGCCCCCACCATGAGCGCTGCTCAGATAGCGATGGTCTGGCCCCTCCTCAGCA   600
                X  R  C  P  H  H  E  R  C  S  D  S  D  G  L  A  P  P  Q  H

                L  I  R  V  E  G  N  L  R  V  E  Y  L  D  D  R  N  T  F  R
Seq_1   579   tcttatccgagtggaaggaaatttgcgtgtggagtatttggatgacagaaacacttttcg   638
              ||||||||||||||||||||||||||||||||||||||||||||||||||||||||||||
Seq_2   601   TCTTATCCGAGTGGAAGGAAATTTGCGTGTGGAGTATTTGGATGACAGAAACACTTTTCG   660
                L  I  R  V  E  G  N  L  R  V  E  Y  L  D  D  R  N  T  F  R

                H  S  V  V  V  P  Y  E  P  P  E  V  G  S  D  C  T  T  I  H
Seq_1   639   acatagtgtggtggtgccctatgagccgcctgaggttggctctgactgtaccaccatcca   698
              ||||||||||||||||||||||||||||||||||||||||||||||||||||||||||||
Seq_2   661   ACATAGTGTGGTGGTGCCCTATGAGCCGCCTGAGGTTGGCTCTGACTGTACCACCATCCA   720
                H  S  V  V  V  P  Y  E  P  P  E  V  G  S  D  C  T  T  I  H

                Y  N  Y  M  C  N  S  S  C  M  G  G  M  N  R  R  P  I  L  T
Seq_1   699   ctacaactacatgtgtaacagttcctgcatgggcggcatgaaccggaggcccatcctcac   758
              ||||||||||||||||||||||||||||||||||||||||||||||||||||||||||||
Seq_2   721   CTACAACTACATGTGTAACAGTTCCTGCATGGGCGGCATGAACCGGAGGCCCATCCTCAC   780
                Y  N  Y  M  C  N  S  S  C  M  G  G  M  N  R  R  P  I  L  T

                                                                     R273L
                I  I  T  L  E  D  S  S  G  N  L  L  G  R  N  S  F  E  V  R
Seq_1   759   catcatcacactggaagactccagtggtaatctactgggacggaacagctttgaggtgcg   818
              ||||||||||||||||||||||||||||||||||||||||||||||||||||||||||||#
Seq_2   781   CATCATCACACTGGAAGACTCCAGTGGTAATCTACTGGGACGGAACAGCTTTGAGGTGCT   840
                I  I  T  L  E  D  S  S  G  N  L  L  G  R  N  S  F  E  V  L

                V  C  A  C  P  G  R  D  R  R  T  E  E  E  N  L  R  K  K  G
Seq_1   819   tgtttgtgcctgtcctgggagagaccggcgcacagaggaagagaatctccgcaagaaagg   878
              ||||||||||||||||||||||||||||||||||||||||||||||||||||||||||||
Seq_2   841   TGTTTGTGCCTGTCCTGGGAGAGACCGGCGCACAGAGGAAGAGAATCTCCGCAAGAAAGG   900
                V  C  A  C  P  G  R  D  R  R  T  E  E  E  N  L  R  K  K  G

                E  P  H  H  E  L  P  P  G  S  T  K  R  A  L  P  N  N  T  S
Seq_1   879   ggagcctcaccacgagctgcccccagggagcactaagcgagcactgcccaacaacaccag   938
              ||||||||||||||||||||||||||||||||||||||||||||||||||||||||||||
Seq_2   901   GGAGCCTCACCACGAGCTGCCCCCAGGGAGCACTAAGCGAGCACTGCCCAACAACACCAG   960
                E  P  H  H  E  L  P  P  G  S  T  K  R  A  L  P  N  N  T  S

                S  S  P  Q  P  K  K  K  P  L  D  G  E  Y  F  T  L  Q  I  R
Seq_1   939   ctcctctccccagccaaagaagaaaccactggatggagaatatttcacccttcagatccg   998
              ||||||||||||||||||||||||||||||||||||||||||||||||||||||||||||
Seq_2   961   CTCCTCTCCCCAGCCAAAGAAGAAACCACTGGATGGAGAATATTTCACCCTTCAGATCCG   1020
                S  S  P  Q  P  K  K  K  P  L  D  G  E  Y  F  T  L  Q  I  R

                G  R  E  R  F  E  M  F  R  E  L  N  E  A  L  E  L  K  D  A
Seq_1   999   tgggcgtgagcgcttcgagatgttccgagagctgaatgaggccttggaactcaaggatgc   1058
              ||||||||||||||||||||||||||||||||||||||||||||||||||||||||||||
Seq_2   1021  TGGGCGTGAGCGCTTCGAGATGTTCCGAGAGCTGAATGAGGCCTTGGAACTCAAGGATGC   1080
                G  R  E  R  F  E  M  F  R  E  L  N  E  A  L  E  L  K  D  A
```

```
                Q  A  G  K  E  P  G  G  S  R  A  H  S  S  H  L  K  S  K  K
Seq_1  1059  ccaggctgggaaggagccaggggggagcagggctcactccagccacctgaagtccaaaaa  1118
             ||||||||||||||||||||||||||||||||||||||||||||||||||||||||||||
Seq_2  1081  CCAGGCTGGGAAGGAGCCAGGGGGGAGCAGGGCTCACTCCAGCCACCTGAAGTCCAAAAA  1140
                Q  A  G  K  E  P  G  G  S  R  A  H  S  S  H  L  K  S  K  K

                G  Q  S  T  S  R  H  K  K  L  M  F  K  T  E  G  P  D  S  D
Seq_1  1119  gggtcagtctacctcccgccataaaaaactcatgttcaagacagaagggcctgactcaga  1178
             ||||||||||||||||||||||||||||||||||||||||||||||||||||||||||||
Seq_2  1141  GGGTCAGTCTACCTCCCGCCATAAAAAACTCATGTTCAAGACAGAAGGGCCTGACTCAGA  1200
                G  Q  S  T  S  R  H  K  K  L  M  F  K  T  E  G  P  D  S  D

                *                             X  X
Seq_1  1179  ctga------------------------------  1182
             ||||##############################
Seq_2  1201  CTGACATTCTCCACTTCTTGTTTCCCCACTGAAA  1234
                *  H  S  P  L  L  V  S  P  L  K
```

C-ES-M

```
Alignment of Sequence_1:  [TP53 CDS.xdna] with  Sequence_2: [Sanger Consensus]
Similarity : 1182/1224 (96,57 %)

                                   M  E  E  P  Q  S  D  P  S  V  E  P  P  L
Seq_1  1     --------------------atggaggagccgcagtcagatcctagcgtcgagccccctc  40
             ####################||||||||||||||||||||||||||||||||||||||||
Seq_2  1     ATGCCTTCCGGGTCACTGCCATGGAGGAGCCGCAGTCAGATCCTAGCGTCGAGCCCCCTC  60
               A  F  R  V  T  A  M  E  E  P  Q  S  D  P  S  V  E  P  P  L

               S  Q  E  T  F  S  D  L  W  K  L  L  P  E  N  N  V  L  S  P
Seq_1  41    tgagtcaggaaacattttcagacctatggaaactacttcctgaaaacaacgttctgtccc  100
             ||||||||||||||||||||||||||||||||||||||||||||||||||||||||||||
Seq_2  61    TGAGTCAGGAAACATTTTCAGACCTATGGAAACTACTTCCTGAAAACAACGTTCTGTCCC  120
               S  Q  E  T  F  S  D  L  W  K  L  L  P  E  N  N  V  L  S  P

               L  P  S  Q  A  M  D  D  L  M  L  S  P  D  D  I  E  Q  W  F
Seq_1  101   ccttgccgtcccaagcaatggatgatttgatgctgtccccggacgatattgaacaatggt  160
             ||||||||||||||||||||||||||||||||||||||||||||||||||||||||||||
Seq_2  121   CCTTGCCGTCCCAAGCAATGGATGATTTGATGCTGTCCCCGGACGATATTGAACAATGGT  180
               L  P  S  Q  A  M  D  D  L  M  L  S  P  D  D  I  E  Q  W  F

                                                               P72R/P
               T  E  D  P  G  P  D  E  A  P  R  M  P  E  A  A  P  P  V  A
Seq_1  161   tcactgaagacccaggtccagatgaagctcccagaatgccagaggctgctccccccgtgg  220
             ||||||||||||||||||||||||||||||||||||||||||||||||||||||:|||||
Seq_2  181   TCACTGAAGACCCAGGTCCAGATGAAGCTCCCAGAATGCCAGAGGCTGCTCCCCSCGTGG  240
               T  E  D  P  G  P  D  E  A  P  R  M  P  E  A  A  P  X  V  A

               P  A  P  A  A  P  T  P  A  A  P  A  P  A  P  S  W  P  L  S
Seq_1  221   cccctgcaccagcagctcctacaccggcggcccctgcaccagcccccctcctggcccctgt  280
             ||||||||||||||||||||||||||||||||||||||||||||||||||||||||||||
Seq_2  241   CCCCTGCACCAGCAGCTCCTACACCGGCGGCCCCTGCACCAGCCCCCTCCTGGCCCCTGT  300
               P  A  P  A  A  P  T  P  A  A  P  A  P  A  P  S  W  P  L  S

               S  S  V  P  S  Q  K  T  Y  Q  G  S  Y  G  F  R  L  G  F  L
Seq_1  281   catcttctgtcccttcccagaaaacctaccagggcagctacggtttccgtctgggcttct  340
             ||||||||||||||||||||||||||||||||||||||||||||||||||||||||||||
Seq_2  301   CATCTTCTGTCCCTTCCCAGAAAACCTACCAGGGCAGCTACGGTTTCCGTCTGGGCTTCT  360
               S  S  V  P  S  Q  K  T  Y  Q  G  S  Y  G  F  R  L  G  F  L

               H  S  G  T  A  K  S  V  T  C  T  Y  S  P  A  L  N  K  M  F
Seq_1  341   tgcattctgggacagccaagtctgtgacttgcacgtactcccctgccctcaacaagatgt  400
             ||||||||||||||||||||||||||||||||||||||||||||||||||||||||||||
Seq_2  361   TGCATTCTGGGACAGCCAAGTCTGTGACTTGCACGTACTCCCCTGCCCTCAACAAGATGT  420
               H  S  G  T  A  K  S  V  T  C  T  Y  S  P  A  L  N  K  M  F

               C  Q  L  A  K  T  C  P  V  Q  L  W  V  D  S  T  P  P  P  G
Seq_1  401   tttgccaactggccaagacctgccctgtgcagctgtgggttgattccacacccccgcccg  460
             ||||||||||||||||||||||||||||||||||||||||||||||||||||||||||||
Seq_2  421   TTTGCCAACTGGCCAAGACCTGCCCTGTGCAGCTGTGGGTTGATTCCACACCCCCGCCCG  480
               C  Q  L  A  K  T  C  P  V  Q  L  W  V  D  S  T  P  P  P  G

               T  R  V  R  A  M  A  I  Y  K  Q  S  Q  H  M  T  E  V  V  R
Seq_1  461   gcacccgcgtccgcgccatggccatctacaagcagtcacagcacatgacggaggttgtga  520
             ||||||||||||||||||||||||||||||||||||||||||||||||||||||||||||
Seq_2  481   GCACCCGCGTCCGCGCCATGGCCATCTACAAGCAGTCACAGCACATGACGGAGGTTGTGA  540
               T  R  V  R  A  M  A  I  Y  K  Q  S  Q  H  M  T  E  V  V  R
```

```
               R  C  P  H  H  E  R  C  S  D  S  D  G  L  A  P  P  Q  H  L
Seq_1  521   ggcgctgcccccaccatgagcgctgctcagatagcgatggtctggcccctcctcagcatc  580
             |||||||||||||||:||||||||||||||||||||||||||||||||||||||||||||
Seq_2  541   GGCGCTGCCCCCACCRTGAGCGCTGCTCAGATAGCGATGGTCTGGCCCCTCCTCAGCATC  600
               R  C  P  H  X  E  R  C  S  D  S  D  G  L  A  P  P  Q  H  L

               I  R  V  E  G  N  L  R  V  E  Y  L  D  D  R  N  T  F  R  H
Seq_1  581   ttatccgagtggaaggaaatttgcgtgtggagtatttggatgacagaaacacttttcgac  640
             ||||||||||||||||||||||||||||||||||||||||||||||||||||||||||||
Seq_2  601   TTATCCGAGTGGAAGGAAATTTGCGTGTGGAGTATTTGGATGACAGAAACACTTTTCGAC  660
               I  R  V  E  G  N  L  R  V  E  Y  L  D  D  R  N  T  F  R  H

               S  V  V  V  P  Y  E  P  P  E  V  G  S  D  C  T  T  I  H  Y
Seq_1  641   atagtgtggtggtgccctatgagccgcctgaggttggctctgactgtaccaccatccact  700
             ||||||||||||||||||||||||||||||||||||||||||||||||||||||||||||
Seq_2  661   ATAGTGTGGTGGTGCCCTATGAGCCGCCTGAGGTTGGCTCTGACTGTACCACCATCCACT  720
               S  V  V  V  P  Y  E  P  P  E  V  G  S  D  C  T  T  I  H  Y

               N  Y  M  C  N  S  S  C  M  G  G  M  N  R  R  P  I  L  T  I
Seq_1  701   acaactacatgtgtaacagttcctgcatgggcggcatgaaccggaggcccatcctcacca  760
             ||||||||||||||||||||||||||||||||||||||||||||||||||||||||||||
Seq_2  721   ACAACTACATGTGTAACAGTTCCTGCATGGGCGGCATGAACCGGAGGCCCATCCTCACCA  780
               N  Y  M  C  N  S  S  C  M  G  G  M  N  R  R  P  I  L  T  I

               I  T  L  E  D  S  S  G  N  L  L  G  R  N  S  F  E  V  R  V
Seq_1  761   tcatcacactggaagactccagtggtaatctactgggacggaacagctttgaggtgcgtg  820
             ||||||||||||||||||||||||||||||||||||||||||||||||||||||||||||
Seq_2  781   TCATCACACTGGAAGACTCCAGTGGTAATCTACTGGGACGGAACAGCTTTGAGGTGCGTG  840
               I  T  L  E  D  S  S  G  N  L  L  G  R  N  S  F  E  V  R  V

               C  A  C  P  G  R  D  R  R  T  E  E  E  N  L  R  K  K  G  E
Seq_1  821   tttgtgcctgtcctgggagagaccggcgcacagaggaagagaatctccgcaagaaagggg  880
             ||||||||||||||||||||||||||||||||||||||||||||||||||||||||||||
Seq_2  841   TTTGTGCCTGTCCTGGGAGAGACCGGCGCACAGAGGAAGAGAATCTCCGCAAGAAAGGGG  900
               C  A  C  P  G  R  D  R  R  T  E  E  E  N  L  R  K  K  G  E

               P  H  H  E  L  P  P  G  S  T  K  R  A  L  P  N  N  T  S  S
Seq_1  881   agcctcaccacgagctgcccccagggagcactaagcgagcactgcccaacaacaccagct  940
             ||||||||||||||||||||||||||||||||||||||||||||||||||||||||||||
Seq_2  901   AGCCTCACCACGAGCTGCCCCCAGGGAGCACTAAGCGAGCACTGCCCAACAACACCAGCT  960
               P  H  H  E  L  P  P  G  S  T  K  R  A  L  P  N  N  T  S  S

               S  P  Q  P  K  K  K  P  L  D  G  E  Y  F  T  L  Q  I  R  G
Seq_1  941   cctctccccagccaaagaagaaaccactggatggagaatatttcacccttcagatccgtg  1000
             ||||||||||||||||||||||||||||||||||||||||||||||||||||||||||||
Seq_2  961   CCTCTCCCCAGCCAAAGAAGAAACCACTGGATGGAGAATATTTCACCCTTCAGATCCGTG  1020
               S  P  Q  P  K  K  K  P  L  D  G  E  Y  F  T  L  Q  I  R  G

               R  E  R  F  E  M  F  R  E  L  N  E  A  L  E  L  K  D  A  Q
Seq_1  1001  ggcgtgagcgcttcgagatgttccgagagctgaatgaggccttggaactcaaggatgccc  1060
             ||||||||||||||||||||||||||||||||||||||||||||||||||||||||||||
Seq_2  1021  GGCGTGAGCGCTTCGAGATGTTCCGAGAGCTGAATGAGGCCTTGGAACTCAAGGATGCCC  1080
               R  E  R  F  E  M  F  R  E  L  N  E  A  L  E  L  K  D  A  Q
```

```
                A  G  K  E  P  G  G  S  R  A  H  S  S  H  L  K  S  K  K  G
Seq_1  1061  aggctgggaaggagccagggggagcagggctcactccagccacctgaagtccaaaaagg  1120
             ||||||||||||||||||||||||||||||||||||||||||||||||||||||||||||
Seq_2  1081  AGGCTGGGAAGGAGCCAGGGGGAGCAGGGCTCACTCCAGCCACCTGAAGTCCAAAAAGG  1140
                A  G  K  E  P  G  G  S  R  A  H  S  S  H  L  K  S  K  K  G

                Q  S  T  S  R  H  K  K  L  M  F  K  T  E  G  P  D  S  D  *
Seq_1  1121  gtcagtctacctcccgccataaaaaactcatgttcaagacagaagggcctgactcagact  1180
             ||||||||||||||||||||||||||||||||||||||||||||||||||||||||||||
Seq_2  1141  GTCAGTCTACCTCCCGCCATAAAAAACTCATGTTCAAGACAGAAGGGCCTGACTCAGACT  1200
                Q  S  T  S  R  H  K  K  L  M  F  K  T  E  G  P  D  S  D  *

                                   X  X
Seq_1  1181  ga----------------------  1182
             ||######################
Seq_2  1201  GACATTCTCCACTTCTGTCCCCCC  1224
                H  S  P  L  L  S  P  X
```

C-ES-P

```
Alignment of Sequence_1:  [TP53 CDS.xdna] with  Sequence_2: [Sanger Consensus]
Similarity : 1180/1223 (96,48 %)

                                M  E  E  P  Q  S  D  P  S  V  E  P  P  L
Seq_1  1      -------------------atggaggagccgcagtcagatcctagcgtcgagccccctct  41
              ###################|||||||||||||||||||||||||||||||||||||||||
Seq_2  1      TGCCTTCCGGGTCACTGCCATGGAGGAGCCGCAGTCAGATCCTAGCGTCGAGCCCCCTCT  60
               A  F  R  V  T  A  M  E  E  P  Q  S  D  P  S  V  E  P  P  L

               S  Q  E  T  F  S  D  L  W  K  L  L  P  E  N  N  V  L  S  P
Seq_1  42     gagtcaggaaacattttcagacctatggaaactacttcctgaaaacaacgttctgtcccc  101
              ||||||||||||||||||||||||||||||||||||||||||||||||||||||||||||
Seq_2  61     GAGTCAGGAAACATTTTCAGACCTATGGAAACTACTTCCTGAAAACAACGTTCTGTCCCC  120
               S  Q  E  T  F  S  D  L  W  K  L  L  P  E  N  N  V  L  S  P

               L  P  S  Q  A  M  D  D  L  M  L  S  P  D  D  I  E  Q  W  F
Seq_1  102    cttgccgtcccaagcaatggatgatttgatgctgtccccggacgatattgaacaatggtt  161
              ||||||||||||||||||||||||||||||||||||||||||||||||||||||||||||
Seq_2  121    CTTGCCGTCCCAAGCAATGGATGATTTGATGCTGTCCCCGGACGATATTGAACAATGGTT  180
               L  P  S  Q  A  M  D  D  L  M  L  S  P  D  D  I  E  Q  W  F

                                                                P72R
               T  E  D  P  G  P  D  E  A  P  R  M  P  E  A  A  P  P  V  A
Seq_1  162    cactgaagacccaggtccagatgaagctcccagaatgccagaggctgctccccccgtggc  221
              ||||||||||||||||||||||||||||||||||||||||||||||||||||#|||||||
Seq_2  181    CACTGAAGACCCAGGTCCAGATGAAGCTCCCAGAATGCCAGAGGCTGCTCCCCGCGTGGC  240
               T  E  D  P  G  P  D  E  A  P  R  M  P  E  A  A  P  R  V  A

               P  A  P  A  A  P  T  P  A  A  P  A  P  A  P  S  W  P  L  S
Seq_1  222    ccctgcaccagcagctcctacaccggcggcccctgcaccagccccctcctggcccctgtc  281
              ||||||||||||||||||||||||||||||||||||||||||||||||||||||||||||
Seq_2  241    CCCTGCACCAGCAGCTCCTACACCGGCGGCCCCTGCACCAGCCCCCTCCTGGCCCCTGTC  300
               P  A  P  A  A  P  T  P  A  A  P  A  P  A  P  S  W  P  L  S

               S  S  V  P  S  Q  K  T  Y  Q  G  S  Y  G  F  R  L  G  F  L
Seq_1  282    atcttctgtcccttcccagaaaacctaccagggcagctacggtttccgtctgggcttctt  341
              ||||||||||||||||||||||||||||||||||||||||||||||||||||||||||||
Seq_2  301    ATCTTCTGTCCCTTCCCAGAAAACCTACCAGGGCAGCTACGGTTTCCGTCTGGGCTTCTT  360
               S  S  V  P  S  Q  K  T  Y  Q  G  S  Y  G  F  R  L  G  F  L

               H  S  G  T  A  K  S  V  T  C  T  Y  S  P  A  L  N  K  M  F
Seq_1  342    gcattctgggacagccaagtctgtgacttgcacgtactcccctgccctcaacaagatgtt  401
              ||||||||||||||||||||||||||||||||||||||||||||||||||||||||||||
Seq_2  361    GCATTCTGGGACAGCCAAGTCTGTGACTTGCACGTACTCCCCTGCCCTCAACAAGATGTT  420
               H  S  G  T  A  K  S  V  T  C  T  Y  S  P  A  L  N  K  M  F

               C  Q  L  A  K  T  C  P  V  Q  L  W  V  D  S  T  P  P  P  G
Seq_1  402    ttgccaactggccaagacctgccctgtgcagctgtgggttgattccacacccccgcccgg  461
              ||||||||||||||||||||||||||||||||||||||||||||||||||||||||||||
Seq_2  421    TTGCCAACTGGCCAAGACCTGCCCTGTGCAGCTGTGGGTTGATTCCACACCCCCGCCCGG  480
               C  Q  L  A  K  T  C  P  V  Q  L  W  V  D  S  T  P  P  P  G

               T  R  V  R  A  M  A  I  Y  K  Q  S  Q  H  M  T  E  V  V  R
Seq_1  462    cacccgcgtccgcgccatggccatctacaagcagtcacagcacatgacggaggttgtgag  521
              ||||||||||||||||||||||||||||||||||||||||||||||||||||||||||||
Seq_2  481    CACCCGCGTCCGCGCCATGGCCATCTACAAGCAGTCACAGCACATGACGGAGGTTGTGAG  540
               T  R  V  R  A  M  A  I  Y  K  Q  S  Q  H  M  T  E  V  V  R
```

```
                  H179R
          R  C  P  H  H  E  R  C  S  D  S  D  G  L  A  P  P  Q  H  L
Seq_1  522   gcgctgcccccaccatgagcgctgctcagatagcgatggtctggcccctcctcagcatct  581
             ||||||||||||||#|||||||||||||||||||||||||||||||||||||||||||||
Seq_2  541   GCGCTGCCCCCACCGTGAGCGCTGCTCAGATAGCGATGGTCTGGCCCCTCCTCAGCATCT  600
          R  C  P  H  R  E  R  C  S  D  S  D  G  L  A  P  P  Q  H  L

          I  R  V  E  G  N  L  R  V  E  Y  L  D  D  R  N  T  F  R  H
Seq_1  582   tatccgagtggaaggaaatttgcgtgtggagtatttggatgacagaaacacttttcgaca  641
             ||||||||||||||||||||||||||||||||||||||||||||||||||||||||||||
Seq_2  601   TATCCGAGTGGAAGGAAATTTGCGTGTGGAGTATTTGGATGACAGAAACACTTTTCGACA  660
          I  R  V  E  G  N  L  R  V  E  Y  L  D  D  R  N  T  F  R  H

          S  V  V  V  P  Y  E  P  P  E  V  G  S  D  C  T  T  I  H  Y
Seq_1  642   tagtgtggtggtgccctatgagccgcctgaggttggctctgactgtaccaccatccacta  701
             ||||||||||||||||||||||||||||||||||||||||||||||||||||||||||||
Seq_2  661   TAGTGTGGTGGTGCCCTATGAGCCGCCTGAGGTTGGCTCTGACTGTACCACCATCCACTA  720
          S  V  V  V  P  Y  E  P  P  E  V  G  S  D  C  T  T  I  H  Y

          N  Y  M  C  N  S  S  C  M  G  G  M  N  R  R  P  I  L  T  I
Seq_1  702   caactacatgtgtaacagttcctgcatgggcggcatgaaccggaggcccatcctcaccat  761
             ||||||||||||||||||||||||||||||||||||||||||||||||||||||||||||
Seq_2  721   CAACTACATGTGTAACAGTTCCTGCATGGGCGGCATGAACCGGAGGCCCATCCTCACCAT  780
          N  Y  M  C  N  S  S  C  M  G  G  M  N  R  R  P  I  L  T  I

          I  T  L  E  D  S  S  G  N  L  L  G  R  N  S  F  E  V  R  V
Seq_1  762   catcacactggaagactccagtggtaatctactgggacggaacagctttgaggtgcgtgt  821
             ||||||||||||||||||||||||||||||||||||||||||||||||||||||||||||
Seq_2  781   CATCACACTGGAAGACTCCAGTGGTAATCTACTGGGACGGAACAGCTTTGAGGTGCGTGT  840
          I  T  L  E  D  S  S  G  N  L  L  G  R  N  S  F  E  V  R  V

          C  A  C  P  G  R  D  R  R  T  E  E  E  N  L  R  K  K  G  E
Seq_1  822   ttgtgcctgtcctgggagagaccggcgcacagaggaagagaatctccgcaagaaagggga  881
             ||||||||||||||||||||||||||||||||||||||||||||||||||||||||||||
Seq_2  841   TTGTGCCTGTCCTGGGAGAGACCGGCGCACAGAGGAAGAGAATCTCCGCAAGAAAGGGGA  900
          C  A  C  P  G  R  D  R  R  T  E  E  E  N  L  R  K  K  G  E

          P  H  H  E  L  P  P  G  S  T  K  R  A  L  P  N  N  T  S  S
Seq_1  882   gcctcaccacgagctgcccccagggagcactaagcgagcactgcccaacaacaccagctc  941
             ||||||||||||||||||||||||||||||||||||||||||||||||||||||||||||
Seq_2  901   GCCTCACCACGAGCTGCCCCCAGGGAGCACTAAGCGAGCACTGCCCAACAACACCAGCTC  960
          P  H  H  E  L  P  P  G  S  T  K  R  A  L  P  N  N  T  S  S

          S  P  Q  P  K  K  K  P  L  D  G  E  Y  F  T  L  Q  I  R  G
Seq_1  942   ctctccccagccaaagaagaaaccactggatggagaatatttcacccttcagatccgtgg  1001
             ||||||||||||||||||||||||||||||||||||||||||||||||||||||||||||
Seq_2  961   CTCTCCCCAGCCAAAGAAGAAACCACTGGATGGAGAATATTTCACCCTTCAGATCCGTGG  1020
          S  P  Q  P  K  K  K  P  L  D  G  E  Y  F  T  L  Q  I  R  G

          R  E  R  F  E  M  F  R  E  L  N  E  A  L  E  L  K  D  A  Q
Seq_1  1002  gcgtgagcgcttcgagatgttccgagagctgaatgaggccttggaactcaaggatgccca  1061
             ||||||||||||||||||||||||||||||||||||||||||||||||||||||||||||
Seq_2  1021  GCGTGAGCGCTTCGAGATGTTCCGAGAGCTGAATGAGGCCTTGGAACTCAAGGATGCCCA  1080
          R  E  R  F  E  M  F  R  E  L  N  E  A  L  E  L  K  D  A  Q
```

```
                A  G  K  E  P  G  G  S  R  A  H  S  S  H  L  K  S  K  K  G
Seq_1  1062  ggctgggaaggagccagggggagcagggctcactccagccacctgaagtccaaaaaggg  1121
             ||||||||||||||||||||||||||||||||||||||||||||||||||||||||||||
Seq_2  1081  GGCTGGGAAGGAGCCAGGGGGAGCAGGGCTCACTCCAGCCACCTGAAGTCCAAAAAGGG  1140
                A  G  K  E  P  G  G  S  R  A  H  S  S  H  L  K  S  K  K  G

                Q  S  T  S  R  H  K  K  L  M  F  K  T  E  G  P  D  S  D  *
Seq_1  1122  tcagtctacctcccgccataaaaaactcatgttcaagacagaagggcctgactcagactg  1181
             ||||||||||||||||||||||||||||||||||||||||||||||||||||||||||||
Seq_2  1141  TCAGTCTACCTCCCGCCATAAAAAACTCATGTTCAAGACAGAAGGGCCTGACTCAGACTG  1200
                Q  S  T  S  R  H  K  K  L  M  F  K  T  E  G  P  D  S  D  *

                                   X  X
Seq_1  1182  a----------------------  1182
             |######################
Seq_2  1201  ACATTCTCCACTTCTGTCCCCCC  1223
                H  S  P  L  L  S  P  X
```

C-ES-Y

Alignment of Sequence_1: [TP53 CDS.xdna] with Sequence_2: [Sanger Consensus]
Similarity : 1181/1242 (95,09 %)

```
                                              M  E  E  P  Q  S  D  P  S  V
Seq_1  1      -------------------------------atggaggagccgcagtcagatcctagcgt  29
              ###############################|||||||||||||||||||||||||||||
Seq_2  1      GCAGCCCCAGACTGCCTTCCGGGTCACTGCCATGGAGGAGCCGCAGTCAGATCCTAGCGT  60
               Q  P  Q  T  A  F  R  V  T  A  M  E  E  P  Q  S  D  P  S  V

               E  P  P  L  S  Q  E  T  F  S  D  L  W  K  L  L  P  E  N  N
Seq_1  30     cgagccccctctgagtcaggaaacattttcagacctatggaaactacttcctgaaaacaa  89
              ||||||||||||||||||||||||||||||||||||||||||||||||||||||||||||
Seq_2  61     CGAGCCCCCTCTGAGTCAGGAAACATTTTCAGACCTATGGAAACTACTTCCTGAAAACAA  120
               E  P  P  L  S  Q  E  T  F  S  D  L  W  K  L  L  P  E  N  N

               V  L  S  P  L  P  S  Q  A  M  D  D  L  M  L  S  P  D  D  I
Seq_1  90     cgttctgtcccccttgccgtcccaagcaatggatgatttgatgctgtccccggacgatat  149
              ||||||||||||||||||||||||||||||||||||||||||||||||||||||||||||
Seq_2  121    CGTTCTGTCCCCCTTGCCGTCCCAAGCAATGGATGATTTGATGCTGTCCCCGGACGATAT  180
               V  L  S  P  L  P  S  Q  A  M  D  D  L  M  L  S  P  D  D  I

               E  Q  W  F  T  E  D  P  G  P  D  E  A  P  R  M  P  E  A  A
Seq_1  150    tgaacaatggttcactgaagacccaggtccagatgaagctcccagaatgccagaggctgc  209
              ||||||||||||||||||||||||||||||||||||||||||||||||||||||||||||
Seq_2  181    TGAACAATGGTTCACTGAAGACCCAGGTCCAGATGAAGCTCCCAGAATGCCAGAGGCTGC  240
               E  Q  W  F  T  E  D  P  G  P  D  E  A  P  R  M  P  E  A  A

               P  P  V  A  P  A  P  A  A  P  T  P  A  A  P  A  P  A  P  S
Seq_1  210    tcccccgtggcccctgcaccagcagctcctacaccggcggcccctgcaccagcccccctc  269
              ||||||||||||||||||||||||||||||||||||||||||||||||||||||||||||
Seq_2  241    TCCCCCGTGGCCCCTGCACCAGCAGCTCCTACACCGGCGGCCCCTGCACCAGCCCCCCTC  300
               P  P  V  A  P  A  P  A  A  P  T  P  A  A  P  A  P  A  P  S

               W  P  L  S  S  S  V  P  S  Q  K  T  Y  Q  G  S  Y  G  F  R
Seq_1  270    ctggcccctgtcatcttctgtcccttcccagaaaacctaccagggcagctacggtttccg  329
              ||||||||||||||||||||||||||||||||||||||||||||||||||||||||||||
Seq_2  301    CTGGCCCCTGTCATCTTCTGTCCCTTCCCAGAAAACCTACCAGGGCAGCTACGGTTTCCG  360
               W  P  L  S  S  S  V  P  S  Q  K  T  Y  Q  G  S  Y  G  F  R

               L  G  F  L  H  S  G  T  A  K  S  V  T  C  T  Y  S  P  A  L
Seq_1  330    tctgggcttcttgcattctgggacagccaagtctgtgacttgcacgtactcccctgccct  389
              ||||||||||||||||||||||||||||||||||||||||||||||||||||||||||||
Seq_2  361    TCTGGGCTTCTTGCATTCTGGGACAGCCAAGTCTGTGACTTGCACGTACTCCCCTGCCCT  420
               L  G  F  L  H  S  G  T  A  K  S  V  T  C  T  Y  S  P  A  L

               N  K  M  F  C  Q  L  A  K  T  C  P  V  Q  L  W  V  D  S  T
Seq_1  390    caacaagatgttttgccaactggccaagacctgccctgtgcagctgtgggttgattccac  449
              ||||||||||||||||||||||||||||||||||||||||||||||||||||||||||||
Seq_2  421    CAACAAGATGTTTTGCCAACTGGCCAAGACCTGCCCTGTGCAGCTGTGGGTTGATTCCAC  480
               N  K  M  F  C  Q  L  A  K  T  C  P  V  Q  L  W  V  D  S  T

               P  P  P  G  T  R  V  R  A  M  A  I  Y  K  Q  S  Q  H  M  T
Seq_1  450    acccccgcccggcacccgcgtccgcgccatggccatctacaagcagtcacagcacatgac  509
              ||||||||||||||||||||||||||||||||||||||||||||||||||||||||||||
Seq_2  481    ACCCCCGCCCGGCACCCGCGTCCGCGCCATGGCCATCTACAAGCAGTCACAGCACATGAC  540
               P  P  P  G  T  R  V  R  A  M  A  I  Y  K  Q  S  Q  H  M  T
```

```
                              C176F
                E   V   V   R   R   C   P   H   H   E   R   C   S   D   S   D   G   L   A   P
Seq_1   510    ggaggttgtgaggcgctgccccccaccatgagcgctgctcagatagcgatggtctggcccc   569
               ||||||||||||||||||#|||||||||||||||||||||||||||||||||||||||||
Seq_2   541    GGAGGTTGTGAGGCGCTTCCCCCACCATGAGCGCTGCTCAGATAGCGATGGTCTGGCCCC   600
                E   V   V   R   R   F   P   H   H   E   R   C   S   D   S   D   G   L   A   P

                P   Q   H   L   I   R   V   E   G   N   L   R   V   E   Y   L   D   D   R   N
Seq_1   570    tcctcagcatcttatccgagtggaaggaaatttgcgtgtggagtatttggatgacagaaa   629
               ||||||||||||||||||||||||||||||||||||||||||||||||||||||||||||
Seq_2   601    TCCTCAGCATCTTATCCGAGTGGAAGGAAATTTGCGTGTGGAGTATTTGGATGACAGAAA   660
                P   Q   H   L   I   R   V   E   G   N   L   R   V   E   Y   L   D   D   R   N

                T   F   R   H   S   V   V   V   P   Y   E   P   P   E   V   G   S   D   C   T
Seq_1   630    cacttttcgacatagtgtggtggtgccctatgagccgcctgaggttggctctgactgtac   689
               ||||||||||||||||||||||||||||||||||||||||||||||||||||||||||||
Seq_2   661    CACTTTTCGACATAGTGTGGTGGTGCCCTATGAGCCGCCTGAGGTTGGCTCTGACTGTAC   720
                T   F   R   H   S   V   V   V   P   Y   E   P   P   E   V   G   S   D   C   T

                T   I   H   Y   N   Y   M   C   N   S   S   C   M   G   G   M   N   R   R   P
Seq_1   690    caccatccactacaactacatgtgtaacagttcctgcatgggcggcatgaaccggaggcc   749
               ||||||||||||||||||||||||||||||||||||||||||||||||||||||||||||
Seq_2   721    CACCATCCACTACAACTACATGTGTAACAGTTCCTGCATGGGCGGCATGAACCGGAGGCC   780
                T   I   H   Y   N   Y   M   C   N   S   S   C   M   G   G   M   N   R   R   P

                I   L   T   I   I   T   L   E   D   S   S   G   N   L   L   G   R   N   S   F
Seq_1   750    catcctcaccatcatcacactggaagactccagtggtaatctactgggacggaacagctt   809
               ||||||||||||||||||||||||||||||||||||||||||||||||||||||||||||
Seq_2   781    CATCCTCACCATCATCACACTGGAAGACTCCAGTGGTAATCTACTGGGACGGAACAGCTT   840
                I   L   T   I   I   T   L   E   D   S   S   G   N   L   L   G   R   N   S   F

                E   V   R   V   C   A   C   P   G   R   D   R   R   T   E   E   E   N   L   R
Seq_1   810    tgaggtgcgtgtttgtgcctgtcctgggagagaccggcgcacagaggaagagaatctccg   869
               ||||||||||||||||||||||||||||||||||||||||||||||||||||||||||||
Seq_2   841    TGAGGTGCGTGTTTGTGCCTGTCCTGGGAGAGACCGGCGCACAGAGGAAGAGAATCTCCG   900
                E   V   R   V   C   A   C   P   G   R   D   R   R   T   E   E   E   N   L   R

                K   K   G   E   P   H   H   E   L   P   P   G   S   T   K   R   A   L   P   N
Seq_1   870    caagaaaggggagcctcaccacgagctgcccccagggagcactaagcgagcactgcccaa   929
               ||||||||||||||||||||||||||||||||||||||||||||||||||||||||||||
Seq_2   901    CAAGAAAGGGGAGCCTCACCACGAGCTGCCCCCAGGGAGCACTAAGCGAGCACTGCCCAA   960
                K   K   G   E   P   H   H   E   L   P   P   G   S   T   K   R   A   L   P   N

                N   T   S   S   S   P   Q   P   K   K   K   P   L   D   G   E   Y   F   T   L
Seq_1   930    caacaccagctcctctccccagccaaagaagaaaccactggatggagaatatttcaccct   989
               ||||||||||||||||||||||||||||||||||||||||||||||||||||||||||||
Seq_2   961    CAACACCAGCTCCTCTCCCCAGCCAAAGAAGAAACCACTGGATGGAGAATATTTCACCCT   1020
                N   T   S   S   S   P   Q   P   K   K   K   P   L   D   G   E   Y   F   T   L

                Q   I   R   G   R   E   R   F   E   M   F   R   E   L   N   E   A   L   E   L
Seq_1   990    tcagatccgtgggcgtgagcgcttcgagatgttccgagagctgaatgaggccttggaact   1049
               ||||||||||||||||||||||||||||||||||||||||||||||||||||||||||||
Seq_2   1021   TCAGATCCGTGGGCGTGAGCGCTTCGAGATGTTCCGAGAGCTGAATGAGGCCTTGGAACT   1080
                Q   I   R   G   R   E   R   F   E   M   F   R   E   L   N   E   A   L   E   L
```

```
               K  D  A  Q  A  G  K  E  P  G  G  S  R  A  H  S  S  H  L  K
Seq_1  1050  caaggatgcccaggctgggaaggagccagggggagcagggctcactccagccacctgaa  1109
             ||||||||||||||||||||||||||||||||||||||||||||||||||||||||||||
Seq_2  1081  CAAGGATGCCCAGGCTGGGAAGGAGCCAGGGGGAGCAGGGCTCACTCCAGCCACCTGAA  1140
               K  D  A  Q  A  G  K  E  P  G  G  S  R  A  H  S  S  H  L  K

               S  K  K  G  Q  S  T  S  R  H  K  K  L  M  F  K  T  E  G  P
Seq_1  1110  gtccaaaaagggtcagtctacctcccgccataaaaaactcatgttcaagacagaagggcc  1169
             ||||||||||||||||||||||||||||||||||||||||||||||||||||||||||||
Seq_2  1141  GTCCAAAAAGGGTCAGTCTACCTCCCGCCATAAAAAACTCATGTTCAAGACAGAAGGGCC  1200
               S  K  K  G  Q  S  T  S  R  H  K  K  L  M  F  K  T  E  G  P

               D  S  D  *                          X  X
Seq_1  1170  tgactcagactga-----------------------------  1182
             |||||||||||||#############################
Seq_2  1201  TGACTCAGACTGACATTCTCCACTTCTTGTTCCCCACTGAAA  1242
               D  S  D  *  H  S  P  L  L  V  P  H  *  X
```

STA-ET-1

Alignment of Sequence_1: [TP53 CDS.xdna]
with Sequence_2: [Sanger Consensus]

```
                               M  E  E  P  Q  S  D  P  S  V  E  P  P  L
Seq_1  1      -------------------atggaggagccgcagtcagatcctagcgtcgagccccctct  41
              ###################|||||||||||||||||||||||||||||||||||||||||
Seq_2  1      TGCCTTCCGGGTCACTGCCATGGAGGAGCCGCAGTCAGATCCTAGCGTCGAGCCCCCTCT  60
               A  F  R  V  T  A  M  E  E  P  Q  S  D  P  S  V  E  P  P  L

               S  Q  E  T  F  S  D  L  W  K  L  L  P  E  N  N  V  L  S  P
Seq_1  42     gagtcaggaaacattttcagacctatggaaactacttcctgaaaacaacgttctgtcccc  101
              ||||||||||||||||||||||||||||||||||||||||||||||||||||||||||||
Seq_2  61     GAGTCAGGAAACATTTTCAGACCTATGGAAACTACTTCCTGAAAACAACGTTCTGTCCCC  120
               S  Q  E  T  F  S  D  L  W  K  L  L  P  E  N  N  V  L  S  P

               L  P  S  Q  A  M  D  D  L  M  L  S  P  D  D  I  E  Q  W  F
Seq_1  102    cttgccgtcccaagcaatggatgatttgatgctgtccccggacgatattgaacaatggtt  161
              ||||||||||||||||||||||||||||||||||||||||||||||||||||||||||||
Seq_2  121    CTTGCCGTCCCAAGCAATGGATGATTTGATGCTGTCCCCGGACGATATTGAACAATGGTT  180
               L  P  S  Q  A  M  D  D  L  M  L  S  P  D  D  I  E  Q  W  F

                                                                  P72R/P
               T  E  D  P  G  P  D  E  A  P  R  M  P  E  A  A  P  P  V  A
Seq_1  162    cactgaagacccaggtccagatgaagctcccagaatgccagaggctgctcccccccgtggc  221
              ||||||||||||||||||||||||||||||||||||||||||||||||||||:||||||
Seq_2  181    CACTGAAGACCCAGGTCCAGATGAAGCTCCCAGAATGCCAGAGGCTGCTCCCCSCGTGGC  240
               T  E  D  P  G  P  D  E  A  P  R  M  P  E  A  A  P  X  V  A

               P  A  P  A  A  P  T  P  A  A  P  A  P  A  P  S  W  P  L  S
Seq_1  222    ccctgcaccagcagctcctacaccggcggcccctgcaccagcccccctcctggcccctgtc  281
              ||||||||||||||||||||||||||||||||||||||||||||||||||||||||||||
Seq_2  241    CCCTGCACCAGCAGCTCCTACACCGGCGGCCCCTGCACCAGCCCCCTCCTGGCCCCTGTC  300
               P  A  P  A  A  P  T  P  A  A  P  A  P  A  P  S  W  P  L  S

               S  S  V  P  S  Q  K  T  Y  Q  G  S  Y  G  F  R  L  G  F  L
Seq_1  282    atcttctgtcccttcccagaaaacctaccagggcagctacggtttccgtctgggcttctt  341
              ||||||||||||||||||||||||||||||||||||||||||||||||||||||||||||
Seq_2  301    ATCTTCTGTCCCTTCCCAGAAAACCTACCAGGGCAGCTACGGTTTCCGTCTGGGCTTCTT  360
               S  S  V  P  S  Q  K  T  Y  Q  G  S  Y  G  F  R  L  G  F  L

               H  S  G  T  A  K  S  V  T  C  T  Y  S  P  A  L  N  K  M  F
Seq_1  342    gcattctgggacagccaagtctgtgacttgcacgtactcccctgccctcaacaagatgtt  401
              ||||||||||||||||||||||||||||||||||||||||||||||||||||||||||||
Seq_2  361    GCATTCTGGGACAGCCAAGTCTGTGACTTGCACGTACTCCCCTGCCCTCAACAAGATGTT  420
               H  S  G  T  A  K  S  V  T  C  T  Y  S  P  A  L  N  K  M  F

               C  Q  L  A  K  T  C  P  V  Q  L  W  V  D  S  T  P  P  P  G
Seq_1  402    ttgccaactggccaagacctgccctgtgcagctgtgggttgattccacacccccgcccgg  461
              ||||||||||||||||||||||||||||||||||||||||||||||||||||||||||||
Seq_2  421    TTGCCAACTGGCCAAGACCTGCCCTGTGCAGCTGTGGGTTGATTCCACACCCCCGCCCGG  480
               C  Q  L  A  K  T  C  P  V  Q  L  W  V  D  S  T  P  P  P  G

               T  R  V  R  A  M  A  I  Y  K  Q  S  Q  H  M  T  E  V  V  R
Seq_1  462    cacccgcgtccgcgccatggccatctacaagcagtcacagcacatgacggaggttgtgag  521
              ||||||||||||||||||||||||||||||||||||||||||||||||||||||||||||
Seq_2  481    CACCCGCGTCCGCGCCATGGCCATCTACAAGCAGTCACAGCACATGACGGAGGTTGTGAG  540
               T  R  V  R  A  M  A  I  Y  K  Q  S  Q  H  M  T  E  V  V  R
```

```
               R  C  P  H  H  E  R  C  S  D  S  D  G  L  A  P  P  Q  H  L
Seq_1  522   gcgctgcccccaccatgagcgctgctcagatagcgatggtctggcccctcctcagcatct  581
             ||||||||||||||||||||||||||||||||||||||||||||||||||||||||||||
Seq_2  541   GCGCTGCCCCCACCATGAGCGCTGCTCAGATAGCGATGGTCTGGCCCCTCCTCAGCATCT  600
               R  C  P  H  H  E  R  C  S  D  S  D  G  L  A  P  P  Q  H  L

               I  R  V  E  G  N  L  R  V  E  Y  L  D  D  R  N  T  F  R  H
Seq_1  582   tatccgagtggaaggaaatttgcgtgtggagtatttggatgacagaaacacttttcgaca  641
             ||||||||||||||||||||||||||||||||||||||||||||||||||||||||||||
Seq_2  601   TATCCGAGTGGAAGGAAATTTGCGTGTGGAGTATTTGGATGACAGAAACACTTTTCGACA  660
               I  R  V  E  G  N  L  R  V  E  Y  L  D  D  R  N  T  F  R  H

               S  V  V  V  P  Y  E  P  P  E  V  G  S  D  C  T  T  I  H  Y
Seq_1  642   tagtgtggtggtgccctatgagccgcctgaggttggctctgactgtaccaccatccacta  701
             ||||||||||||||||||||||||||||||||||||||||||||||||||||||||||||
Seq_2  661   TAGTGTGGTGGTGCCCTATGAGCCGCCTGAGGTTGGCTCTGACTGTACCACCATCCACTA  720
               S  V  V  V  P  Y  E  P  P  E  V  G  S  D  C  T  T  I  H  Y

               N  Y  M  C  N  S  S  C  M  G  G  M  N  R  R  P  I  L  T  I
Seq_1  702   caactacatgtgtaacagttcctgcatgggcggcatgaaccggaggcccatcctcaccat  761
             ||||||||||||||||||||||||||||||||||||||||||||||||||||||||||||
Seq_2  721   CAACTACATGTGTAACAGTTCCTGCATGGGCGGCATGAACCGGAGGCCCATCCTCACCAT  780
               N  Y  M  C  N  S  S  C  M  G  G  M  N  R  R  P  I  L  T  I

               I  T  L  E  D  S  S  G  N  L  L  G  R  N  S  F  E  V  R  V
Seq_1  762   catcacactggaagactccagtggtaatctactgggacggaacagctttgaggtgcgtgt  821
             ||||||||||||||||||||||||||||||||||||||||||||||||||||||||||||
Seq_2  781   CATCACACTGGAAGACTCCAGTGGTAATCTACTGGGACGGAACAGCTTTGAGGTGCGTGT  840
               I  T  L  E  D  S  S  G  N  L  L  G  R  N  S  F  E  V  R  V

               C  A  C  P  G  R  D  R  R  T  E  E  E  N  L  R  K  K  G  E
Seq_1  822   ttgtgcctgtcctgggagagaccggcgcacagaggaagagaatctccgcaagaaagggga  881
             ||||||||||||||||||||||||||||||||||||||||||||||||||||||||||||
Seq_2  841   TTGTGCCTGTCCTGGGAGAGACCGGCGCACAGAGGAAGAGAATCTCCGCAAGAAAGGGGA  900
               C  A  C  P  G  R  D  R  R  T  E  E  E  N  L  R  K  K  G  E

               P  H  H  E  L  P  P  G  S  T  K  R  A  L  P  N  N  T  S  S
Seq_1  882   gcctcaccacgagctgcccccagggagcactaagcgagcactgcccaacaacaccagctc  941
             ||||||||||||||||||||||||||||||||||||||||||||||||||||||||||||
Seq_2  901   GCCTCACCACGAGCTGCCCCCAGGGAGCACTAAGCGAGCACTGCCCAACAACACCAGCTC  960
               P  H  H  E  L  P  P  G  S  T  K  R  A  L  P  N  N  T  S  S

               S  P  Q  P  K  K  K  P  L  D  G  E  Y  F  T  L  Q  I  R  G
Seq_1  942   ctctccccagccaaagaagaaaccactggatggagaatatttcacccttcagatccgtgg  1001
             ||||||||||||||||||||||||||||||||||||||||||||||||||||||||||||
Seq_2  961   CTCTCCCCAGCCAAAGAAGAAACCACTGGATGGAGAATATTTCACCCTTCAGATCCGTGG  1020
               S  P  Q  P  K  K  K  P  L  D  G  E  Y  F  T  L  Q  I  R  G

               R  E  R  F  E  M  F  R  E  L  N  E  A  L  E  L  K  D  A  Q
Seq_1  1002  gcgtgagcgcttcgagatgttccgagagctgaatgaggccttggaactcaaggatgccca  1061
             ||||||||||||||||||||||||||||||||||||||||||||||||||||||||||||
Seq_2  1021  GCGTGAGCGCTTCGAGATGTTCCGAGAGCTGAATGAGGCCTTGGAACTCAAGGATGCCCA  1080
               R  E  R  F  E  M  F  R  E  L  N  E  A  L  E  L  K  D  A  Q
```

```
                A  G  K  E  P  G  G  S  R  A  H  S  S  H  L  K  S  K  K  G
Seq_1  1062  ggctgggaaggagccagggggggagcagggctcactccagccacctgaagtccaaaaaggg  1121
             ||||||||||||||||||||||||||||||||||||||||||||||||||||||||||||
Seq_2  1081  GGCTGGGAAGGAGCCAGGGGGGAGCAGGGCTCACTCCAGCCACCTGAAGTCCAAAAAGGG  1140
                A  G  K  E  P  G  G  S  R  A  H  S  S  H  L  K  S  K  K  G

                Q  S  T  S  R  H  K  K  L  M  F  K  T  E  G  P  D  S  D  *
Seq_1  1122  tcagtctacctcccgccataaaaaactcatgttcaagacagaagggcctgactcagactg  1181
             ||||||||||||||||||||||||||||||||||||||||||||||||||||||||||||
Seq_2  1141  TCAGTCTACCTCCCGCCATAAAAAACTCATGTTCAAGACAGAAGGGCCTGACTCAGACTG  1200
                Q  S  T  S  R  H  K  K  L  M  F  K  T  E  G  P  D  S  D  *

                                  X  X
Seq_1  1182  a----------------------  1182
             |######################
Seq_2  1201  ACATTCTCCACTTCTGTCCCCCC  1223
                H  S  P  L  L  S  P  X
```

SK-N-MC

```
Alignment of Sequence_1:  [TP53 mRNA.xdna]
with  Sequence_2: [SK-N-MC_TP53_seq_rev.fasta]

Seq_1  1     ctcaaaagtctagagccaccgtccagggagcaggtagctgctgggctccggggacacttt  60
             ##||||||||||||||||||||||||||||||||||||||||||||||||||||||||||
Seq_2  288   --CAAAAGTCTAGAGCCACCGTCCAGGGAGCAGGTAGCTGCTGGGCTCCGGGGACACTTT  231


Seq_1  61    gcgttcgggctgggagcgtgctttccacgacggtgacacgcttccctggattggcagcca  120
             ||||||||||||||||||||||||||||||||||||||||||||||||||||||######
Seq_2  230   GCGTTCGGGCTGGGAGCGTGCTTTCCACGACGGTGACACGCTTCCCTGGATTGG------  177

                                        CDS
Seq_1  121   gactgccttccgggtcactgccatggaggagccgcagtcagatcctagcgtcgagccccc  180
             ############################################################
Seq_2  176   ------------------------------------------------------------  177


Seq_1  181   tctgagtcaggaaacattttcagacctatggaaactacttcctgaaaacaacgttctgtc  240
             ############################################################
Seq_2  176   ------------------------------------------------------------  177


Seq_1  241   ccccttgccgtcccaagcaatggatgatttgatgctgtccccggacgatattgaacaatg  300
             ############################################################
Seq_2  176   ------------------------------------------------------------  177


Seq_1  301   gttcactgaagacccaggtccagatgaagctcccagaatgccagaggctgctccccccgt  360
             ############################################################
Seq_2  176   ------------------------------------------------------------  177


Seq_1  361   ggcccctgcaccagcagctcctacaccggcggcccctgcaccagccccctcctggcccct  420
             ############################################################
Seq_2  176   ------------------------------------------------------------  177


Seq_1  421   gtcatcttctgtcccttcccagaaaacctaccagggcagctacggtttccgtctgggctt  480
             ############################################################
Seq_2  176   ------------------------------------------------------------  177


Seq_1  481   cttgcattctgggacagccaagtctgtgacttgcacgtactcccctgccctcaacaagat  540
             #####################################|||||||||||||||||||||||
Seq_2  176   -------------------------------------TACTCCCCTGCCCTCAACAAGAT  154


Seq_1  541   gttttgccaactggccaagacctgccctgtgcagctgtgggttgattccacacccccgcc  600
             ||||||||||||||||||||||||||||||||||||||||||||||||||||||||||||
Seq_2  153   GTTTTGCCAACTGGCCAAGACCTGCCCTGTGCAGCTGTGGGTTGATTCCACACCCCCGCC  94


Seq_1  601   cggcacccgcgtccgcgccatggccatctacaagcagtcacagcacatgacggaggttgt  660
             ||||||||||||||||||||||||||||||||||||||||||||||||||||||||||||
Seq_2  93    CGGCACCCGCGTCCGCGCCATGGCCATCTACAAGCAGTCACAGCACATGACGGAGGTTGT  34


Seq_1  661   gaggcgctgcccccaccatgagcgctgctcagatagcgatggtctggcccctcctcagca  720
             |||||||||||||||||||||||||||||||||###########################
Seq_2  33    GAGGCGCTGCCCCCACCATGAGCGCTGCTCAGA---------------------------  1
```
